# Two-layered blood-lipid phantom and method to determine absorption and oxygenation employing changes in moments of DTOFs

**DOI:** 10.1364/BOE.492168

**Published:** 2023-06-21

**Authors:** Aleh Sudakou, Heidrun Wabnitz, André Liemert, Martin Wolf, Adam Liebert

**Affiliations:** 1 Nałęcz Institute of Biocybernetics and Biomedical Engineering Polish Academy of Sciences, Warsaw, Poland; 2 Physikalisch-Technische Bundesanstalt (PTB), Berlin, Germany; 3 Institut für Lasertechnologien in der Medizin und Meßtechnik an der Universität Ulm, Germany; 4 Department of Neonatology, University Hospital Zurich, University of Zurich, Zurich, Switzerland

## Abstract

Near-infrared spectroscopy (NIRS) is an established technique for measuring tissue oxygen saturation (StO_2_), which is of high clinical value. For tissues that have layered structures, it is challenging but clinically relevant to obtain StO_2_ of the different layers, e.g. brain and scalp. For this aim, we present a new method of data analysis for time-domain NIRS (TD-NIRS) and a new two-layered blood-lipid phantom. The new analysis method enables accurate determination of even large changes of the absorption coefficient (Δ*µ*_a_) in multiple layers. By adding Δ*µ*_a_ to the baseline *µ*_a_, this method provides absolute *µ*_a_ and hence StO_2_ in multiple layers. The method utilizes (i) changes in statistical moments of the distributions of times of flight of photons (DTOFs), (ii) an analytical solution of the diffusion equation for an N-layered medium, (iii) and the Levenberg–Marquardt algorithm (LMA) to determine Δ*µ*_a_ in multiple layers from the changes in moments. The method is suitable for NIRS tissue oximetry (relying on *µ*_a_) as well as functional NIRS (fNIRS) applications (relying on Δ*µ*_a_). Experiments were conducted on a new phantom, which enabled us to simulate dynamic StO_2_ changes in two layers for the first time. Two separate compartments, which mimic superficial and deep layers, hold blood-lipid mixtures that can be deoxygenated (using yeast) and oxygenated (by bubbling oxygen) independently. Simultaneous NIRS measurements can be performed on the two-layered medium (variable superficial layer thickness, *L*), the deep (homogeneous), and/or the superficial (homogeneous). In two experiments involving ink, we increased the nominal *µ*_a_ in one of two compartments from 0.05 to 0.25 cm^−1^, *L* set to 14.5 mm. In three experiments involving blood (*L* set to 12, 15, or 17 mm), we used a protocol consisting of six deoxygenation cycles. A state-of-the-art multi-wavelength TD-NIRS system measured simultaneously on the two-layered medium, as well as on the deep compartment for a reference. The new method accurately determined *µ*_a_ (and hence StO_2_) in both compartments. The method is a significant progress in overcoming the contamination from the superficial layer, which is beneficial for NIRS and fNIRS applications, and may improve the determination of StO_2_ in the brain from measurements on the head. The advanced phantom may assist in the ongoing effort towards more realistic standardized performance tests in NIRS tissue oximetry. Data and MATLAB codes used in this study were made publicly available.

## 1. Introduction

Near-infrared spectroscopy (NIRS) is a non-invasive and safe (even for long continuous monitoring) technique in biomedical optics for studying brain functions and its disorders. NIRS tissue oximetry enables monitoring vital parameters in real time, reporting on the balance between oxygen supply and consumption. The clinical usefulness of NIRS has been demonstrated by a wide range of clinical applications and trials (reviewed in [1–9]) and the popularity of NIRS continues to grow in part due to the continuous advancements in instrumentation and the resultant increasing number of NIRS systems (reviewed in [10–15]). A significant improvement to NIRS tissue oximetry would be the determination of StO_2_ in multiple layers and a means of testing such method. Another field of NIRS is functional NIRS (fNIRS), which typically involves small hemodynamic changes (i.e. small Δ*µ*_a_) related to brain activity [16,17]. The new data analysis method and the phantom introduced in this study may be useful for both fNIRS and NIRS tissue oximetry applications.

The analysis of NIRS measurements carried out on a layered tissue structure, such as the head, require a multi-layered model as the homogeneous model is inadequate [18–22]. Recent advances in modeling the light propagation in tissue (theories, solutions, and validations) are explained in the most recent book by Martelli et al. [23]. In this study, we used the time-domain NIRS (TD-NIRS) modality, which records the distribution of times of flight of photons (DTOFs), providing the most information about light propagation compared to continuous-wave (steady-state) and frequency-domain modalities [16]. Light in tissue can be modelled using the radiative transfer equation (RTE) and a commonly employed solution is the diffusion equation, which relies on the diffusion approximation [24–26]. Helton et al. [27] presented an open-access numerical routine for solving the diffusion equation for an arbitrary number of layers, for the steady-state case and in the time domain. Geiger et al. [28] modeled light propagation using spherical harmonics instead of the diffusion equation and presented the analytical solutions for the RTE. To solve for complex geometries, the finite element method can be used, e.g. NIRFAST [29]. Another approach for modelling light propagation is based on the Monte Carlo method [30,31], which allows implementing any geometry but the main disadvantage is the long computational time. We will use the analytical solution for an N-layered medium presented by Liemert et al. [32], which applies the Laplace transform (LT) to a solution in the frequency domain (could be either numerical or analytical) to obtain the solution in the time domain. LT has important advantages over the commonly used Fourier transform, such as accuracy, efficiency (speed-up of up to several orders of magnitude), and numerical stability. Having a model for light propagation and its solution, the optical properties can be obtained from measured NIRS data by solving the inverse problem.

Various algorithms have been developed for determining the absorption (*µ*_a_) or its changes (Δ*µ*_a_) at multiple depths of a multi-layered turbid medium using TD-NIRS measurements, e.g. more recently in Refs. [28,33–36]. We will use the Levenberg–Marquardt algorithm (LMA), which allows solving nonlinear iterative least squares minimization problems [37,38]. The LMA is a fitting routine that searches for a solution that minimizes an error, i.e. the difference between the measured data and the theoretically-calculated data. Garcia et al. [35] proposed implementing the Kalman filter algorithm, which is another iterative algorithm that has some advantages over the LMA. Instead of using the DTOF as a measurand, Hallacoglu et al. [39] used the amplitude change and the phase shift at multiple source-detector distances as the measurands for the LMA. Steinbrink et al. [40] used changes in the number of photons in different time-channels of a DTOF for determining Δ*µ*_a_ at different depths, since different time-channels have different depth sensitivity profiles. Similarly, different-order statistical moments of the DTOF have different depth sensitivity profiles and Liebert et al. [41,42] outlined the use of moments for the determination of Δµ_a_ at multiple depths. This has become a well-established method, e.g. [43–46]. Recent performance-assessment studies, including quantitative tests, found that moments outperform other measurands in terms of assessing Δ*µ*_a_ in deep layers [47–49]. Using changes in moments at multiple wavelengths, which have slightly different depth sensitivity profiles, it was possible to estimate changes in the concentration of an injected indocyanine green (ICG) bolus in up to 24 layers [50]. However, up to now, the method relied on the assumption that the changes in moments are linearly proportional to Δ*µ*_a_, where the proportionality constants are the sensitivity factors [41]. This inverse problem of determining Δ*µ*_a_ from changes in moments was solved using the linear least squares method or the singular value decomposition approach, i.e. non-iterative approaches.

One goal of this work is to improve the method based on the analysis of moments of DTOFs by using the analytical solution of the diffusion equation for a multi-layered medium and implementing the LMA. With this approach, we solve a nonlinear iterative least squares minimization problem, hence we remove the limiting assumption that changes in moments are linearly proportional to Δ*µ*_a_. In addition to testing the method on phantom measurements, we will investigate how errors in the values of the baseline parameters affect the determined Δ*µ*_a_ values. We aim to provide a more accurate and robust method for determining Δ*µ*_a_ at multiple depths in a multi-layered medium.

When introducing a new method of data analysis or a new system, phantom measurements are a necessary test before conducting *in-vivo* studies as phantoms allow to study the influence of the instrumental factors and uncertainties for well-defined geometries and optical properties [51]. There is an ongoing effort towards standardization of system performance tests in the field of NIRS [15,51], which has become increasingly important in part due to the increasing number of NIRS systems [10–15]. Recently, Lanka et al. [52] presented the results of the BitMap campaign, which was a multi-laboratory effort to perform a standardized performance assessment of instruments for diffuse optics, which used the BIP [53], nEUROPt [54], and MEDPHOT [55] protocols. Other standardization efforts resulted in a consensus by many groups on the guidelines for the best practices for fNIRS publications [56]. Over the past decade, many tissue-mimicking phantoms for optical spectroscopy have been developed, and they are reviewed in [51,57–59], including challenges related to their construction and utilization. Konugolu Venkata Sekar et al. [60] developed solid tissue-like phantoms that mimic the anatomically correct 3D shapes of human organs for biomedical applications, e.g. [61]. For testing tissue oximeters and for other biophotonics applications, there is a need for advanced phantoms that can provide realistic simulations of tissues’ optical properties. Liu et al. [62] developed a phantom that achieves simulating vascular oxygenation and perfusion, by filling with blood the tubes that mimic vessels. Afshari et al. [59] developed a phantom with an array of 148 cylindrical channels, filled with whole bovine blood, as a new approach for standardized testing of clinical oximeters. An established concept for a phantom is using whole human blood (mixed in a turbid suspension) for mimicking repeatable controlled dynamic oxygen saturation (StO_2_) levels from 0 to 100%. To date, all presented phantoms that used this concept had a single homogeneous liquid compartment, e.g. [63–67]. However, when NIRS is intended to study deeper structures, e.g. the brain, it is crucial to address the influence of the superficial layer on the measured StO_2_ [18–22].

The second goal of this work is to develop a phantom with two compartments (superficial and deep), each of which can hold a mixture containing blood that can be oxygenated or deoxygenated. The phantom is a tool that will allow quantitatively studying the contamination from the superficial layer when determining StO_2_ in a two-layered medium. We will utilize the phantom to demonstrate the proposed method’s capabilities of determining StO_2_ in both layers.

Data and MATLAB codes used in this study were made publicly available [68].

## 2. Methods and materials

### 2.1 NIRS system

The multi-wavelength TD-NIRS system was developed by Gerega et al. [69] at IBIB PAN and it was described and experimentally tested in our recent study [70], revealing its advantages compared to the current state-of-the-art systems. The system can simultaneously acquire DTOFs at 16 spectral channels (from 680 to 875 nm, with a 12.5 nm/channel width) for each of two detection modules. Each module consists of a polychromator, a multi-anode 16-channel photomultiplier tube, and a 16-channel TCSPC card, as described in [70]. Two emitting fibers and two detection fiber bundles allow simultaneous measurements on two windows of the phantom in reflectance geometry. The previous supercontinuum laser (SC480-8 WhiteLase, Fianium, UK) was replaced with a newer laser (NKT SuperK EXTREME (EXR-15), Denmark), which has similar characteristics. We used the repetition rate of 78 MHz and set the constant current power to 50% in the laser driver. The parameters of a TCSPC card were configured so that each DTOF contained 1024 time bins with a width of 12.22 ps. This width is sufficiently small as a study on accuracy suggested that a bin width of about 10 ps enables a better than 5% accuracy in quantifying optical properties using the curve fitting method [71].

In this study, the distance between the source and detector fibers was set to 30 mm. The acquisition time was 0.3 s and the sampling rate was 3 Hz. To increase the signal-to-noise ratio and reduce the computational time, 9 consecutive DTOFs were summed for measurements on phantoms with blood, resulting in a sampling time of 3 s. For measurements with ink, 128 DTOFs were summed for each Δ*µ*_a_ step, resulting in a sampling time of 42.7 s. We used a shorter sampling time for analyzing measurements with blood to resolve the dynamic changes of StO_2_. We used a longer sampling time for experiments involving ink to reduce noise since the optical properties were stable.

To measure the instrumental response function (IRF), an emission fiber was aligned with a detection fiber bundle in transmission geometry at a distance of 6 cm with a neutral density filter between them and two pieces of white paper covering the source fiber and the detection fiber bundle [72]. The maximum laser power was measured as 15 mW for each emission fiber, which would equate to a power density of less than 1.5 mW/mm^2^ on a skin's surface [70]. For a recent comprehensive study on IRF, see the study conducted by Pirovano et al. [73].

### 2.2 Data analysis

The measured NIRS data were stored and later analyzed using custom codes written in MATLAB R2020b. The LMA was implement using the function *lsqcurvefit* in MATLAB.

#### 2.2.1 Determination of *µ*_a_ and *µ*′_s_ (homogeneous model)

To determine the values of *µ*_a_ and the reduced scattering coefficient (*µ*′_s_) from a measured DTOF, we used a solution of the diffusion equation for a homogeneous medium and the LMA. This is known as the curve-fitting method and it is regarded as the gold-standard approach for analyzing time-domain diffuse reflectance measurements [37,38,71], and an example of the distribution of error norm was presented in [74].

We used a homogeneous semi-infinite medium model under the extrapolated boundary conditions, which was done by using the solution of the diffusion equation for an N-layered medium [32] with N set to 1. We used equations presented in [25], which allow to account for the boundary conditions. The theoretically-obtained DTOFs are convolved with the IRF that was measured at the start of an experiment. Analyzing each measured DTOF separately, we first subtracted the background signal and then used the time-channels from 85% on the rising edge to 1% on the tail for determining *µ*_a_ and *µ*′_s_.

#### 2.2.2 Determination of Δ*µ*_a_ in two layers

In this section we outline an improved method of determining Δ*µ*_a_ in multiple layers. To obtain the absolute *µ*_a_ with this method, we added the determined Δ*µ*_a_ values to the baseline *µ*_a_ values, which were obtained using the method explained in the previous section.

DTOF is represented by a histogram of photon counts (*N_i_*) at time channels (*t_i_*) indexed by *i* [41]. The first three statistical moments of DTOFs are: $m_{0}=N_{\text{tot}}=\sum N_{i}$ (total number of photons), $m_{1}=\sum t_{i}N_{i}/N_{\text{tot}}$ (mean time of flight), and $m_{2}^{C}=V=\sum {\left(t_{i}-m_{1}\right)}^{2}N_{i}/N_{\text{tot}}$ (variance). The change in attenuation is: $\Delta A=-\log(N_{\text{tot}}^{*}/N_{\text{tot}})$ , where $N_{\text{tot}}^{*}$ is after Δ*µ*_a_. Moments have several important advantageous properties. To deconvolve the influence of the IRF, the moments of the IRF are subtracted from the moments of the DTOF; valid for $m_{1}$ , $m_{2}^{C}$ , and $m_{3}^{C}$ (third central moment) [46]. The resulting moments can be used to calculate the optical properties of tissue [75]. Changes in moments are independent of the IRF under the assumption that the moments of the IRF are constant. The uncertainty of moments due to photon noise, which behaves like Poisson noise (Poisson statistics), can be calculated using higher-order moments [42,46].

The method that uses the changes in moments (Δ*A*, Δ*m*_1_, and/or Δ*V*) requires two DTOFs: during baseline and after Δ*µ*_a_. For small Δ*µ*_a_ in multiple layers, the corresponding changes in moments are linearly proportional to Δ*µ*_a_ in each layer, where the proportionality constants are the sensitivity factors (SF) for each layer, as explained by Steinbrink et al. [40] and Liebert et al. [41,42]. The SF for baseline optical properties can be obtained using Monte Carlo simulations [30] or an analytical solution of the diffusion equation [43], and then it is a linear inverse problem to determine Δ*µ*_a_ from the measured changes in moments.

To improve the method, we overcome the assumption that the changes in moments are linearly proportional to Δ*µ*_a_ by using the solution of the diffusion equation for an N-layered medium [32] and the LMA. In this study, we set N to 2, assumed the deeper layer is semi-infinite, and assumed the extrapolated boundary conditions. The use of LMA avoids the need for sensitivity factors. For a guess of Δ*µ*_a_ in each layer, the analytical solution is used to generate the corresponding two DTOFs for which changes in moments are calculated. The LMA searches for Δ*µ*_a_ in each layer that result in the smallest error norm (χ^2^), which is the difference between the measured and the theoretically calculated changes in moments. The different-order moments have different units and levels of uncertainty. To account for these, we weighted each moment by its uncertainty due to photon noise [42]. When all three moments are considered, χ^2^ can be calculated using the equation:

(1) $$\chi^{2}={\left(\frac{\Delta A_{\text{sim}}-\Delta A}{\delta \Delta A}\right)}^{2}+{\left(\frac{\Delta m_{1,\text{sim}}-\Delta m_{1}}{\delta \Delta m_{1}}\right)}^{2}+{\left(\frac{\Delta V_{\text{sim}}-\Delta V}{\delta \Delta V}\right)}^{2}$$

where changes in moments are calculated from two DTOFs: $\Delta A=A-A^{*}$ , $\Delta m_{1}=m_{1}-m_{\text{1}}^{*}$ , and $\Delta V=V-V^{*}$ , where the star denotes the value of a moment after Δ*µ*_a_. For a guess of Δ*µ*_a_, the corresponding changes in moments ( $\Delta A_{\text{sim}}$ , $\Delta m_{\text{1,sim}}$ , and $\Delta V_{\text{sim}}$ ) are calculated from the theoretically obtained DTOFs. The uncertainties due to photon noise ( δΔA , $\delta \Delta m_{1}$ , and δΔV ) can be calculated using higher-order moments [42]. We make a simplifying assumption that the noise of moments of two DTOFs (before and after Δ*µ*_a_) is the same (true if Δ*µ*_a_ = 0).

(2) $$\chi^{2}={\left(\frac{\Delta A_{\text{sim}}-\Delta A}{\sqrt{2/N_{\text{tot}}}}\right)}^{2}+{\left(\frac{\Delta m_{1,\text{sim}}-\Delta m_{1}}{\sqrt{2V/N_{\text{tot}}}}\right)}^{2}+{\left(\frac{\Delta V_{\text{sim}}-\Delta V}{\sqrt{2(m_{4}^{C}-V^{2})/N_{\text{tot}}}}\right)}^{2}$$

(3) $$\frac{2}{N_{\text{tot}}}\chi^{2}={\left(\Delta A_{\text{sim}}-\Delta A\right)}^{2}+{\left(\frac{\Delta m_{1,\text{sim}}-\Delta m_{1}}{\sqrt{V}}\right)}^{2}+{\left(\frac{\Delta V_{\text{sim}}-\Delta V}{\sqrt{m_{4}^{C}-V^{2}}}\right)}^{2}$$

To assess the method, we will calculate and plot the distribution of $(2/N_{\text{tot}})\chi^{2}$ (Eq. (3)) for all combinations of Δ*µ*_a_ in two layers [34,74,76,77]. *N*_tot_ is a scaling factor, and it is a function of the selected laser intensity and acquisition time. Therefore, the calculated χ^2^ is unitless, except for the linear dependence on *N*_tot_.

In this study, we did not use the intensity information (Δ*A*) for determining Δ*µ*_a_ because it requires careful data processing to account for possible laser drifts and changes in the optical filters. During measurements, the optical filters were adjusted (by rotating the Neutral Density filter) in order to regulate the count rate. Additionally, Δ*A* is more susceptible to laser drifts than other moments.

We used the developed phantom, which mimics two layers with changing *µ*_a_, for experimentally testing the new method. The method requires knowing the values of all parameters at baseline, which can be obtained for phantom measurements. The refractive index (*n*) was assumed to be 1.33 for both layers because the main component of the phantom mixture was distilled water. The thickness of the superficial layer (*L*) was measured with rulers glued to the phantom. To obtain the values of *µ*_a_ and *µ*′_s_ of each layer, we used measurements on the individual compartments (superficial and deep) and applied the curve-fitting method with the homogeneous model. For experiments involving blood, the targeted optical properties at baseline were the same for both layers, so measurements on a single layer were sufficient to determine the optical properties of both layers.

Although the changes in the first three statistical moments are independent of the IRF, their values are influenced by the chosen time-channels that are used for their computation [43]. We computed moments using the same time-channels for all theoretically obtained and measured DTOFs: from 25% on the rising edge to 3% on the tail of the DTOF that was measured at baseline. Prior to calculations of moments, the theoretically obtained DTOFs were convolved with the IRF that was measured at the start of an experiment.

The new method’s computation time for each DTOF is typically a few seconds, which can be reduced with a good initial guess. Therefore, we employed the established method based on moments, which uses sensitivity factors, for obtaining the initial guess of Δ*µ*_a_ in two layers. We obtained the sensitivity factors by computing the changes in moments resulting from ±2% Δ*µ*_a_ in each layer, as done in [43].

#### 2.2.3 Calculation of concentrations of oxy- and deoxyhemoglobin

To determine the concentrations of oxyhemoglobin (HbO_2_) and deoxyhemoglobin (Hb) in experiments involving blood, we used the determined *µ*_a_ values at multiple wavelengths and the known molar absorption coefficients (ε) of HbO_2_ and Hb. We used 9 spectral channels (3^rd^ to 11^th^ out of 16) covering the spectral range from 705 to 805 nm, which is consistent with our previous study with blood-lipid phantoms [70]; the dependence on the choice of wavelengths should be further investigated.

We used the ε spectra provided by Gratzer et al. [78] and the absorption spectra of water provided by Matcher et al. [79]. We calculated the concentrations of only HbO_2_ and Hb using the *µ*_a_ values, which were obtained by subtracting the *µ*_a_ of water (multiplied by 0.985, which was the volume fraction of water and SMOFlipid) from the determined *µ*_a_ values.

### 2.3 Phantom setup

#### 2.3.1 Construction of phantom

In recent years, various tissue-mimicking phantoms containing blood have been developed, e.g. [59,62–67]. However, to our knowledge, no presented phantom has two blood-lipid compartments with variable StO_2_, which could be used to mimic the extra- and intracerebral compartments of the head.

**Fig. 1.**
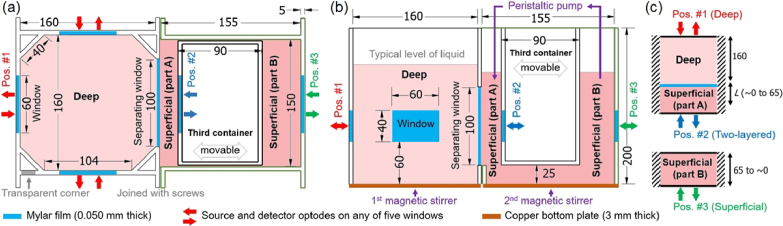
Diagrams of the phantom showing top view (a) and side view (b), and the corresponding model (c). The three containers are labeled: Deep, Superficial, and Third container, which is movable. Units are mm.

We present a new tissue-mimicking phantom that was built in-house at IBIB PAN. The phantom consists of three containers, as shown in Fig. 1. **The deep container** is similar to the one presented by Kleiser et al. [63] and holds the liquid for the deep compartment. The container has an irregular octagonal shape. Three walls have windows for optodes, which allow three NIRS systems to measure simultaneously, and the fourth wall has a separating window (100 mm × 100 mm). **The superficial container** holds the liquid for the superficial compartment and **the third container** separates this liquid into parts A and B. The superficial container has three walls and the side without a wall attaches to the deep container. The third container is shorter and suspended, which leaves a 25 mm gap underneath for the liquid to flow between parts A and B, as shown in Fig. 1 (b). The thickness of part A can be changed from approximately 0 to 65 mm by moving the third container. Part B of the superficial compartment allows adding and efficiently mixing ingredients. Measurements can be performed simultaneously on any of the five windows (60 mm × 40 mm) for measuring on different compartments: the deep (Pos. #1), the two-layered medium (Pos. #2), and the superficial (Pos. #3). Below we list the main features of the phantom.

**Materials**. Five walls have windows for measurements (next to red, blue, and green arrows in Fig. 1) and these walls are made from black PVC (5 mm thick). All other walls and the covering lids are made from transparent PVC (5 mm thick), which allows seeing inside the phantom. To see and measure the height of the liquid in the deep container, one corner was cut out and replaced with transparent PVC, shown in Fig. 1 (a). To prevent ambient light interference during measurements, the entire phantom was covered with a black blanket. The deep and the superficial containers are held together by four screws, which have washers (2 mm thick solid plates cut from PVC), and in-between is a glued water-resistant rubber cord, which gets squeezed to prevent leakage. The bottom parts are made from copper (3 mm thick), which allows using hot plates for controlling the temperature. Each of the five windows (60 × 40 mm) has two latches for holding the optode holders (58 × 38 mm), which were 3D printed (pure black ABS material, Zortrax Z-ABS v2). All windows were covered with translucent Mylar film (0.050 mm thick, 785-0786 RS Pro, UK), which was permanently fixed to the walls using silicone. The effect of a thin, slightly scattering Mylar film on diffuse reflectance measurements can be considered negligible [80]. To limit ambient oxygen diffusion, the two containers were covered with lids, which have openings for fixing a thermometer, an oxygen supply, a peristaltic pump, and for adding ingredients.

**Thickness of the superficial layer (*L*)**. The third container is designed to slide along one direction, which is achieved by four linear bearing guides attached to its top and two linear shafts attached to the top of the other two containers. A threaded rod is attached between the two linear shafts to enable precise movement of the container. Rulers were glued on top of the third and the superficial containers to facilitate measurement of the third container’s position and hence *L*. To counteract buoyant forces, heavy objects can be placed inside the third container.

**Separating window and bending of Mylar film**. To separate mixtures contained in the deep and superficial containers, we covered the separating window (100 mm x 100 mm, as shown in Fig. 1) with Mylar film. When the Mylar film gets wet, it is able to bend easily. To maintain a constant position of the Mylar film, we applied pressure on it by pouring more liquid into the deep container, so that the liquid level was approximately 2 cm higher than in the superficial container. This created a uniform high pressure on the Mylar film, resulting in a constant and repeatable bending. During data analysis, we estimated that the Mylar film bent by approximately 0.5 mm, which we approximated as a flat boundary, given the large area of the separating window.

**Stirring and heating**. Stirring and heating were accomplished by placing the deep and the superficial containers on top of two hot plates (Stuart US152D, Bibby Sterlin Ltd. and SMP-TC, SET GSM, Poland). In all experiments, the stirring was set at 500 RPM. Additionally, in the superficial container, a peristaltic pump (372.C Elpan, Poland) was used to move the liquid from the top of part B to the top of part A (shown in Fig. 1 (b)) at a flow rate of 500 ml per min through an 8 mm diameter silicone tube. In experiments involving blood, the temperature was maintained at 37 ± 1 °C. To verify the efficacy of stirring, we added ink to either the deep or the superficial container and checked how quickly the measured signals reached new stable values, which typically occurred within about 10 seconds.

**Controlled and monitored parameters**. In experiments involving blood, the following parameters were controlled and monitored. The temperature in each compartment was recorded every 10 s using two submerged digital thermometers (RC-5+, Elitech). The pH level was kept constant at 7.4 by adding the phosphate-buffered saline powder (PBS powder, sc-24947, ChemCruz). The blood was deoxygenated by adding dry bakery yeast (Drozdze Suszone Instant, Dr. Oetker, Poland). To oxygenate the blood, medical-grade oxygen from a tank was bubbled (3 L/min flow setting on the pressure regulator) through an air stone (Mist-Air, fine bubbles, Kordon) submerged in the deep container. The air stone was held in place by a long rigid plastic tube and positioned in the middle of the deep container, approximately 3 cm above the bottom. Experiments involving ink required controlling only the stirring rate.

**Simultaneous NIRS measurements** can be performed on up to five windows, i.e. with up to five NIRS systems. If the source-detector distance (ρ) of the optodes fixed on the phantom is 40 mm, then the minimum distance between fibers on different windows is at least 75 mm, as in [63]. Light has a negligible chance of traversing 75 mm if the optical properties of the phantom are similar to those of biological tissue [76].

**Boundary conditions**. We assumed semi-infinite model with extrapolated boundary conditions. We approximated the deep container as an infinite medium (semi-infinite for measurements on the surface). For ρ *=* 40 mm, the distance from a fiber to the closest neighbouring wall is 60 mm, so the laser light needs to traverse at least 160 mm to reach a neighbouring wall and then a detection fiber bundle. Optodes were attached 75 mm above the ground level and the liquid was filled up to about 150 mm (or 170 mm) above the ground level in the superficial (or deep) container. We assumed that black PVC and black optode holders absorb all light that penetrates the surface. We neglected the effect of the Mylar film [80].

#### 2.3.2 Characterizing ink and SMOFlipid

The use of ink and Intralipid in tissue-simulating phantoms has been studied and described in the literature, e.g. Di Ninni et al. [81,82] and Spinelli et al. [83,84]. In this section, we summarize how we determined the nominal values of *µ*_a_ and *µ′*_s_, which we used as a guide for the experimental protocol and later for a comparison with our TD-NIRS results.

We used SMOFlipid 20% (Fresenius Kabi, Poland) as a substitute for Intralipid, which we confirmed has similar *µ′*_s_ and *µ*_a_. We calculated the nominal *µ′*_s_ of a phantom based on the volume fraction of SMOFlipid [81,84]:

(4) $$\mu_{\text{s}}^{\prime }=\frac{V_{\text{SL}}}{V_{\text{Diluted ink}}+V_{\text{SL}}+V_{\text{water}}}\varepsilon_{\text{s},\text{SL}}^{\prime }$$

where $\varepsilon_{\text{s},\text{SL}}^{\prime }$ is the specific reduced scattering coefficient of SMOFlipid. We used the value 21.5 mm^-1^ for 750 nm, which was reported for Intralipid [81,83,84]. *V*_Dilted ink_, *V*_SL_, and *V*_water_ are the volumes of diluted ink, SMOFlipid, and water, respectively.

We characterized the *µ*_a_ of ink (Rotring, black, 23 ml, Germany) because it can have large brand-to-brand and batch-to-batch variations [82,84]. We diluted pure ink in distilled water (*d*_ink_ *=* weight of ink / total weight), measured its absorbance (A_p_) in a 1 cm thick cuvette with a spectrophotometer, and calculated its effective absorption coefficient (*ε*_eff,ink_ *=* A_p_ / *d*_ink_). At 750 nm, we obtained *ε*_eff,ink_ *=* 626 mm^-1^, which is consistent with a previously reported value for this brand of ink [82]. We combined the value of *ε*_eff,ink_ obtained from spectrophotometric measurements and the value of the single-scattering albedo (Λ) to predict the value of the specific absorption coefficient of ink (*ε*_a,ink_), as proposed in [84]. By definition, Λ *= *(*ε*_eff,ink_ *– ε*_a,ink_) / *ε*_eff,ink_ and we used the value of 0.15 for Λ. We calculated the nominal *µ*_a_ of a phantom based on the volume fraction of the diluted ink [82,84]:

(5) $$\mu_{\text{a,nominal}}=\mu_{\text{a,water}}+\frac{V_{\text{Diluted ink}}d_{\text{ink}}}{V_{\text{Diluted ink}}+V_{\text{SL}}+V_{\text{water}}}\varepsilon_{\text{eff,ink}}(1-\Lambda )$$

We made a simplifying assumption that SMOFlipid has the same *µ*_a_ as water, since their *µ*_a_ are similar and the main constituent of the phantoms in all experiments was water.

#### 2.3.3 Protocol for experiments involving ink

We conducted two experiments involving ink, in which we increased *µ*_a_ in either the deep or the superficial compartment. In one compartment, the targeted *µ*_a_ values ranged from 0.05 to 0.25 cm^-1^ (at 750 nm) in 20 steps, while in the other compartment, the targeted *µ*_a_ was fixed at 0.10 cm^-1^ at 750 nm. The targeted *µ′*_s_ was 10.7 cm^-1^ at 750 nm in both compartments. These values of optical properties are within the typical range for tissues [85,86], specifically of the human head [87]. Table 1 lists the targeted amounts of ingredients. To increase *µ*_a_ without changing *µ′*_s_, we prepared Mixture #2 (water, diluted ink, and SMOFlipid), which has a much higher *µ*_a_ (∼ 4.7 cm^-1^) but the same *µ′*_s_ as the mixture in the phantom. We set *L* to 14.5 mm in both experiments, which were conducted on two consecutive days. We used the TD-NIRS system to measure on the deep compartment (Pos. #1), the two-layered medium (Pos. #2), and/or the superficial compartment (Pos.#3), according to Fig. 1.

**Table 1.** Ingredients (in grams) and targeted *µ*_a_ values (at 750 nm) for the two experiments involving ink. SL is SMOFlipid.

Exp.	Deep container				Superficial container			
	Water	SL	Mixture #2	Targeted µ_a_ / cm^-1^	Water	SL	Mixture #2	Targeted µ_a_ / cm^-1^
#1	3420	180	17 → 170	0.05 → 0.25	1710	90	27	0.10
#2	-//-	-//-	54	0.10	-//-	-//-	8.5 → 85	0.05 → 0.25

The nominal *µ*_a_ values were calculated by using the weights of the added ingredients and Eq. (5). Distilled water and SMOFlipid were weighted with a precision of 0.2 g. For adding Mixture #2, we used a syringe and measured its weight before and after injecting the mixture into the phantom, with a precision of 0.1 µg. We waited at least 40 s for the ingredients to stir before taking NIRS measurements. After adding an ingredient, its measured weight was recorded and used to update the targeted weight of the next ingredient to be added, which helped achieve the targeted *µ*_a_ values across the twenty Δ*µ*_a_ steps.

#### 2.3.4 Protocol for experiments involving blood

We conducted three experiments involving blood using the same protocol, but setting different thickness of the superficial layer *L* (12, 15, and 17 mm). We used TD-NIRS system to measure on the deep compartment (Pos. #1) and the two-layered medium (Pos. #2). Measurements on the deep compartment allow assessing the repeatability of the experiment, as they are independent of *L*.

We changed the oxygen saturation (StO_2_) of blood separately in the superficial and the deep compartments, in one compartment at a time. The targeted *µ′*_s_ of both compartments was 10.7 cm^-1^ at 750 nm. Table 2 lists the protocol steps along with the targeted StO_2_. Table 3 lists the targeted amounts of ingredients. The preparation of the phantom before starting TD-NIRS measurements comprised of six steps (P1 to P6). After step P6, the deep compartment contained 3366 g of distilled water with PBS and 180 g of SMOFlipid, as in Table 3. In step S1, we added human erythrocyte concentrate to each compartment. We used a syringe with 1.6 mm inner diameter for handling blood-containing mixtures. In step S2, we took some of the mixture (approximately 40 ml) from the deep compartment, mixed yeast in it, and poured it back into the deep compartment. We denoted this as time zero (Time *=* 0 min). In step S3, we bubbled oxygen in the deep compartment for 2 min. In step S4, we added yeast to the superficial compartment using a similar procedure as was used for adding yeast to the deep compartment. Then, we repeated step S3 three times (steps S5, S6, and S7). The protocol consisted of six deoxygenation cycles: one in the superficial compartment and five in the deep compartment. These cycles are indicated by six arrows at the top of figures that show time traces of the TD-NIRS results.

**Table 2.** Protocol steps and the targeted StO_2_ in two compartments for the three experiments involving blood.

Step	Time/min	Description	Targeted StO_2_ / %	
			Deep	Superficial
P1		Turn on the laser and let it warm up for at least 1 hour		
P2		Assemble three containers and add water with PBS to both compartments		
P3		Start stirring and heating, and attach two thermometers		
P4		Remove air bubbles in both compartments by tapping with a long metal rod		
P5		Add SMOFlipid to both compartments, which makes them opaque		
P6		Measure IRF and then start measuring on the phantom		
S1		Add blood to each compartment	∼100	∼100
S2	0	Add yeast to the Deep compartment		
		Wait 13 min until plateau (cycle #1)	100 → 0	
S3	13–15	Oxygenate the Deep compartment	0 → 100	
		Wait 15 min until plateau (cycle #2)	100 → 0	
S4	31	Add yeast to the Superficial compartment		
		Wait 15 min until plateau (cycle #3)		100 → 0
S5	45–47	Oxygenate the Deep compartment	0 → 100	
		Wait 13 min until plateau (cycle #4)	100 → 0	
S6	60–62	Oxygenate the Deep compartment	0 → 100	
		Wait 15 min until plateau (cycle #5)	100 → 0	
S7	77–79	Oxygenate the Deep compartment	0 → 100	
		Wait 13 min until plateau (cycle #6)	100 → 0	

**Table 3.** Ingredients for each of the three experiments involving blood. SL is SMOFlipid.

Container	PBS (water) after step P2	SL after step P5	Blood after step S1	Yeast after steps S2 and S4	Total (excluding yeast)
Deep	3366 g	180 g	54 g	5.4 g	3600 g
Superficial	1683 g	90 g	27 g	2.7 g	1800g
Percentage	93.5%	5%	1.5%	0.1%	100%

The three experiments were conducted on different days. The first experiment (Exp. #1, *L* *=* 12 mm) and third experiment (Exp. #3, *L* *=* 17 mm) used blood from the same blood transfusion bag. The second experiment (Exp. #2, *L* = 15 mm) used another blood transfusion bag.

## 3. Results

### 3.1 Method for determining Δ $\mu_{a}$ in two layers

**Fig. 2.**
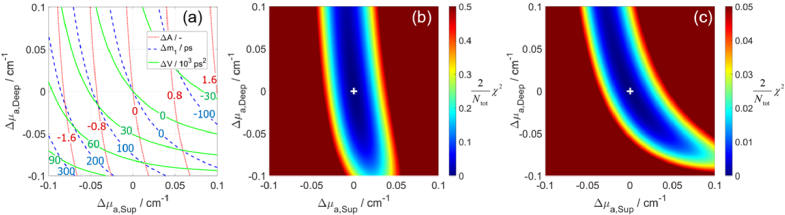
Changes in moments (Δ*A*, Δ*m*_1_, and Δ*V*) for all combinations of Δ*µ*_a,Sup_ and Δ*µ*_a,Deep_ (a). Error norm (χ^2^) for all combinations of Δ*µ*_a,Sup_ and Δ*µ*_a,Deep_ (white cross marks true values) when using changes in three moments (b) or using only Δ*m*_1_ and Δ*V* (c). Baseline parameters are *µ*_a,Sup_ = *µ*_a,Deep_ = 0.13 cm^-1^, *µ′*_s_ = 10 cm^-1^, *L* = 12 mm, and *n* = 1.33.

Here we examine the proposed method for determining Δ*µ*_a_ in two layers. We calculated the values of Δ*A*, Δ*m*_1_, and Δ*V* for all combinations of Δ*µ*_a,Sup_ and Δ*µ*_a,Deep_, and the results are presented in Fig. 2 (a), where the changes in each statistical moment are shown using contour lines. The lines of all pairs of two moments intersect only once, which confirms that there is a unique solution of Δ*µ*_a,Sup_ and Δ*µ*_a,Deep_ when changes of any two moments are analyzed. The slopes of such lines (changes in moments versus Δ*µ*_a,Sup_ and Δ*µ*_a,Deep_) are related to depth selectivity [48,88], which is a metric that reflects the sensitivity of a measurand to Δ*µ*_a,Deep_ in relation to Δ*µ*_a,Sup_. The lines for Δ*A* are more vertical, indicating that Δ*A* is more sensitive to Δ*µ*_a,Sup_ compared to Δ*µ*_a,Deep_, i.e. Δ*A* has low depth selectivity. The lines become more horizontal for higher-order moments, corresponding to increased depth selectivity, which is a well-known property of moments [48,88]. For small values of Δ*µ*_a_, the lines for all moments be approximated as linear functions. However, for larger values of Δ*µ*_a_, the lines become increasingly nonlinear, confirming the need for using the LMA, i.e. solving a nonlinear problem of determining Δ*µ*_a_ in multiple layers using changes in moments.

We calculated the error norm (χ^2^) using Eq. (3) for all combinations of Δ*µ*_a,Sup_ and Δ*µ*_a,Deep_. The distribution of χ^2^ is shown in Fig. 2 (b) when all three moments are considered and in Fig. 2 (c) when excluding the term with Δ*A*, i.e. using only the terms containing Δ*m*_1_ and Δ*V* in Eq. (3). The presented χ^2^ distributions have a single minimum, i.e. no local minima, confirming the suitability of the LMA approach for finding this minimum. We verified that the LMA reaches the same solution (determined Δ*µ*_a_ in two layers) regardless of the initial guess.

The shape of the χ^2^ distribution depends on which moments are used and the weights assigned to them. When three moments are used (Fig. 2 (b)), the contribution from Δ*A* dominates, resulting in a χ^2^ distribution that is more dependent on the superficial layer compared to the deep layer. This distribution resembles the line where Δ*A* *=* 0 in Fig. 2 (a). When the term with Δ*A* is excluded (Fig. 2 (c)), the contribution from Δ*m*_1_ becomes dominant, and the shape of the resulting χ^2^ distribution resembles the line where Δ*m*_1_ *=* 0 in Fig. 2 (a). These results are consistent with the differences in magnitudes of the contrast-to-noise ratio (CNR), e.g. reported in [88] where a 15% increase in *µ*_a,Sup_ resulted in a CNR of about 100 for Δ*N*_tot_, 30 for Δ*m*_1_, and 10 for Δ*V*. Therefore, for a typical Δ*µ*_a_, the term with Δ*A* in Eq. (3) contributes much more to the value of χ^2^ than the term with Δ*m*_1_, which is consistent with the one-order magnitude difference in the colorbars for χ^2^ in Fig. 2 (b) and (c). If only a single moment is considered in Eq. (3), the shape of the χ^2^ distribution would follow the moment’s zero contour line shown in Fig. 2 (a) and the value of χ^2^ would be zero along this line, resulting in a lack of a single minimum.

**Fig. 3.**
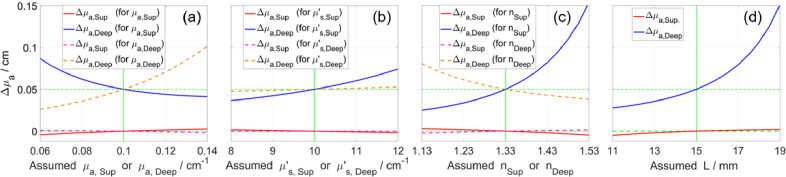
Determined Δ*µ*_a,Sup_ and Δ*µ*_a,Deep_ for different assumed values of baseline parameters: *µ*_a_ (a), *µ′*_s_ (b), *n* (c), and *L* (d). Green lines mark true values: Δ*µ*_a,Sup_ = 0 and Δ*µ*_a,Deep_ = 0.05 cm^-1^, *µ*_a_ = 0.1 cm^-1^, *µ′*_s_ = 10 cm^-1^, *L* = 15 mm, and *n* = 1.33.

We examined the impact of an error in the value of a baseline parameter on the determined Δ*µ*_a_ and the results are presented in Fig. 3. We simulated two DTOFs (at baseline and after Δ*µ*_a,Deep_ = 0.05 cm^-1^) and retrieved Δ*µ*_a,Sup_ and Δ*µ*_a,Deep_ but using different assumed values for one of the baseline parameters: *µ*_a_, *µ′*_s_, and *n* in the superficial or the deep compartment, as well as *L*. The findings depend on the chosen ground truth values of all parameters (for both simulated DTOFs), but the results in Fig. 3 are a representative example. Figure 3 (b) shows that *µ*′_s,Deep_ has little effect on TD-NIRS signals, which was also found in previous studies [89]. Figure 3 (a, b) demonstrates that an error in the assumed value of *µ*_a,Sup_ has an opposite effect on the determined values of Δ*µ*_a_, compared to an error in the assumed value of *µ*′_s,Sup_. This agrees with the general understanding that these parameters (*µ*_a_ and *µ*′_s_) have contrasting impacts on the shape of the DTOF [74]. Errors in *µ*′_s,Sup_ and *n*_Sup_ have similar effects (Fig. 3 (b, c)), which is expected as both impact how quickly light passes through a medium. The determined Δ*µ*_a,Sup_ is minimally affected by *L* (Fig. 3 (d)). However, when *L* is underestimated (or overestimated), then the determined Δ*µ*_a,Deep_ approaches (or moves away from) the true value of Δ*µ*_a,Sup_. This dependence on *L* remained consistent when using ground truth values of Δ*µ*_a,Sup_ *=* 0.05 cm^-1^ and Δ*µ*_a,Deep_ *=* 0.

### 3.2 Experiments involving ink

**Fig. 4.**
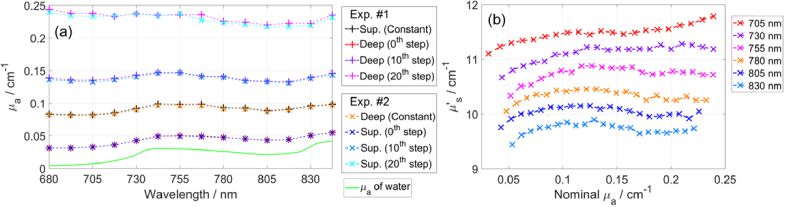
(a) Determined *µ*_a_ for two experiments involving ink, for three nominal *µ*_a_ (0.05, 0.15, and 0.25 cm^-1^ at 750 nm). (b) Determined *µ′*_s_ for 20 Δ*µ*_a_ steps for Exp. #1. Results were obtained using measurements on each compartment (Pos. #1 or #3 in Fig. 1), analyzed using the curve-fitting method with a homogeneous model.

Figure 4 (a) shows the determined *µ*_a_ for two experiments involving ink, for all wavelengths, for both compartments, for three Δ*µ*_a_ steps. The *µ*_a_ values for both experiments are similar and consistent with the targeted *µ*_a_ values in Table 1, which demonstrates good control and repeatability of experiments. The spectral shape of *µ*_a_ for the smallest nominal *µ*_a_ resembles the shape of the *µ*_a_ of water. For the spectral shape of *µ′*_s_, please refer to Fig. 8 (b).

Figure 4 (b) shows the determined *µ′*_s_ values for 20 Δ*µ*_a_ steps in the deep compartment in Exp. #1, at different wavelengths. For both experiments, the *µ′*_s_ sometimes slightly increase with *µ*_a_, especially for the first few (about four) Δ*µ*_a_ steps. However, for most wavelengths, the values of *µ′*_s_ changed by less than approximately 0.3 cm^-1^ (less than 3%), following a 0.20 cm^−1^ (500%) increase in *µ*_a_. One cause may be the coupling of Δ*µ*_a_ to Δ*µ′*_s_, which we previously reported for our system [70] by measuring on a well-characterized array of solid homogeneous phantoms that have the same *µ′*_s_ (either 5, 10, 15, or 20 cm^−1^) and different *µ*_a_ values (ranging from 0 to 0.35 cm^-1^) [55], as part of the BitMap campaign [52]. For a comparison of the coupling with other systems, see [52]. Other causes for changes in *µ′*_s_ could be, e.g. possible timing drifts in the laser output. Increasing the concentration of ink should not have influenced the *µ′*_s_ because we used Mixture #2 (containing water, SMOFlipid, and ink) that had the same *µ′*_s_ as the phantom but much higher *µ*_a_ (refer to section 2.3.3).

**Fig. 5.**
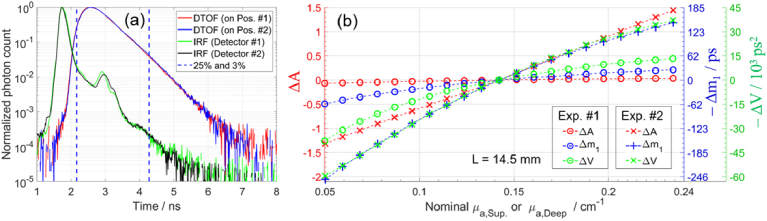
(a) Normalized IRFs and DTOFs (when targeted *µ*_a_ *=* 0.10 cm^-1^ at 750 nm in both compartments), for two source-detector pairs on Pos. #1 and #2, at 768 nm. (b) Changes in moments (Δ*A*, Δ*m*_1_, and Δ*V*) for 20 Δ*µ*_a_ steps, measured on Pos. #2 (two-layered), at 768 nm.

**Fig. 6.**
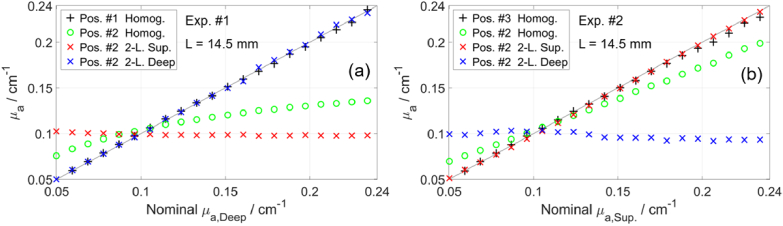
Determined *µ*_a_ for 20 Δ*µ*_a_ steps in first (a) and second (b) experiments involving ink, at 768 nm. Measurements on a compartment with increasing *µ*_a_ (Pos. #1 or Pos. #3) were analyzed using the curve-fitting method with a homogeneous model (black crosses). Measurements on two-layered medium (Pos. #2) were analyzed using the same method (green circles) and the method based on changes in moments with a two-layered model (red and blue crosses).

Figures 5 and 6 present results at 768 nm (8^th^ spectral channel) and similar results were obtained at other wavelengths. Figure 5 (a) shows the normalized IRFs and DTOFs for the two source-detector pairs located on Pos. #1 and #2, when the targeted *µ*_a_ was 0.10 cm^-1^ at 750 nm in both compartments. The two source-detector pairs had similar IRFs and hence similar DTOFs. The total number of photons in the DTOF (*N*_tot_) was between approximately 5.4 to 0.5 million photons at 768 nm for different Δ*µ*_a_ steps, with lower values of *N*_tot_ at higher *µ*_a_.

Figure 5 (b) shows the changes in moments (Δ*A*, Δ*m*_1_, and Δ*V*) resulting from 20 Δ*µ*_a_ steps. The baseline was selected as the middle measurement (10^th^ Δ*µ*_a_ step, when the targeted *µ*_a_ = 0.15 cm^-1^ at 750 nm). All moments show high contrast to Δ*µ*_a,Sup_ and much lower contrast to Δ*µ*_a,Deep_, which is related to the large *L* (14.5 mm). The contrast to Δ*µ*_a,Deep_ (and hence depth selectivity) increases for higher-order moments, consistent with the results in Fig. 2 (a). Notably, ΔA appears to be close to zero even for the largest Δ*µ*_a,Deep_. However, to assess a measurand’s capabilities of detecting Δ*µ*_a_, it is essential to consider also the contrast-to-noise ratio (CNR) [48,88].

Figure 6 presents the determined *µ*_a_ for 20 Δ*µ*_a_ steps for the two experiments involving ink. One source-detector pair was used to measure on the compartment in which *µ*_a_ was changed: the deep compartment for Exp. #1 and the superficial compartment for Exp. #2. These measurements were analyzed with the curve-fitting method (homogeneous model) to determine *µ*_a_ and *µ′*_s_. The determined *µ*_a_ values (black crosses in Fig. 6) are close to the nominal *µ*_a_ values (the identity line) for all 20 Δ*µ*_a_ steps and for both experiments. The signal level (i.e. *N*_tot_) decreased for higher *µ*_a_, leading to increased noise, which could be the reason for the slight discrepancies for higher *µ*_a_. For measurements on a two-layered medium (Pos. #2), the curve-fitting method (homogeneous model) provided *µ*_a_ values (green circles in Fig. 6) that were between the *µ*_a_ values of the two compartments and closer to the superficial compartment’s *µ*_a_. This highlights the need for a two-layered model when measuring on a two-layered medium. We applied the proposed method based on the changes in moments to determine Δ*µ*_a,Sup_ and Δ*µ*_a,Deep_, and added to them the baseline *µ*_a_ values. The resulting values of *µ*_a,Sup_ and *µ*_a,Deep_ (red and blue crosses in Fig. 6) are close to the nominal values for all 20 Δ*µ*_a_ steps. These results are comparable to the results of similar experiments that were reported in e.g. [33,36,43]. Possible drifts in the laser output could affect the moments, which may account for the slight deviations from a horizontal line for the determined *µ*_a,Deep_ in Exp. #2 (blue crosses in Fig. 6 (b)). Another cause could be the *µ′*_s_ changes shown in Fig. 4 (b), which can affect only the results for the deep layer in Exp. 2, based on the findings in Fig. 3 (b).

### 3.3 Experiments involving blood

**Fig. 7.**
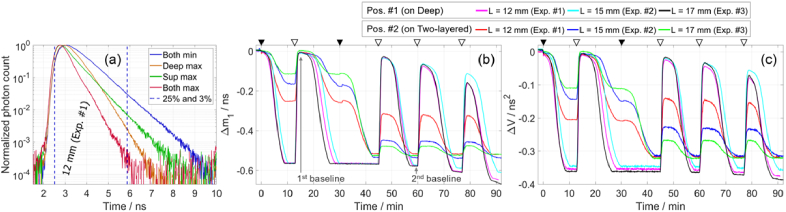
(a) DTOFs measured on Pos. #2 at different *µ*_a_ in two compartments, at 705 nm. Changes in moments, Δ*m*_1_ (b) and Δ*V* (c), measured on Pos. #1 and #2, for three experiments involving blood. Filled triangles indicate when yeast was added, separately in deep and superficial compartments, and hollow triangles denote the start of bubbling oxygen in the deep compartment (these triangles are also shown in Fig. 9 to 12).

**Fig. 8.**
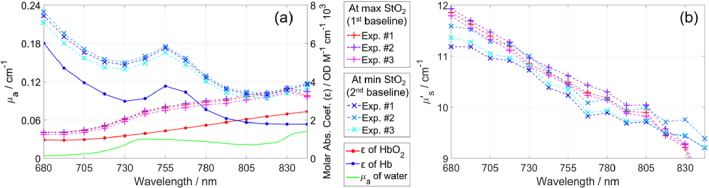
Determined *µ*_a_ (a) and *µ*′_s_ (b) at maximum and minimum StO_2_ for three experiments involving blood, measured on the deep compartment. The molar absorption coefficients (ε) [78] and the *µ*_a_ of water [79] were used for calculating the concentrations of HbO_2_ and Hb.

**Fig. 9.**
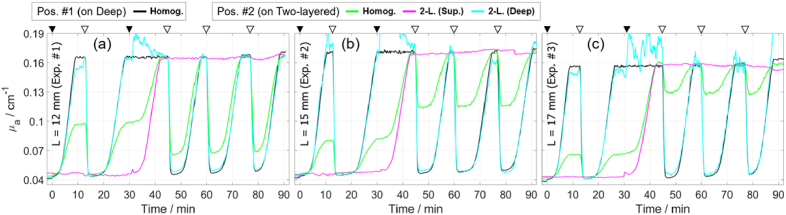
Determined *µ*_a_ at 705 nm in first (a), second (b), and third (c) experiments involving blood. Measurements were analyzed using the curve-fitting method with a homogeneous model (Homog.) and using the changes in moments with a two-layered model (2-L). For visualization, a moving average window (10 data points) was applied to all curves. Triangles at the top are explained in caption of Fig. 7.

**Fig. 10.**
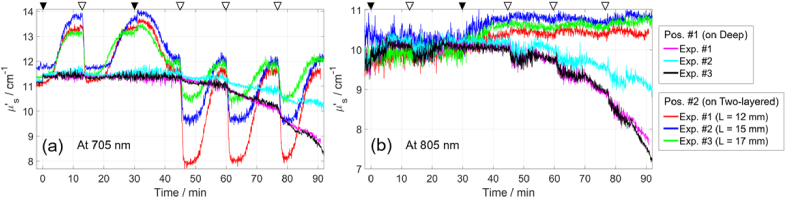
Reduced scattering coefficient *µ*′_s_ in three experiments involving blood, at 705 nm (a) and 805 nm (b). Measurements were analyzed using the curve-fitting method with a homogeneous model and the corresponding determined *µ*_a_ values are black and green curves in Fig. 9. Triangles at the top are explained in caption of Fig. 7.

**Fig. 11.**
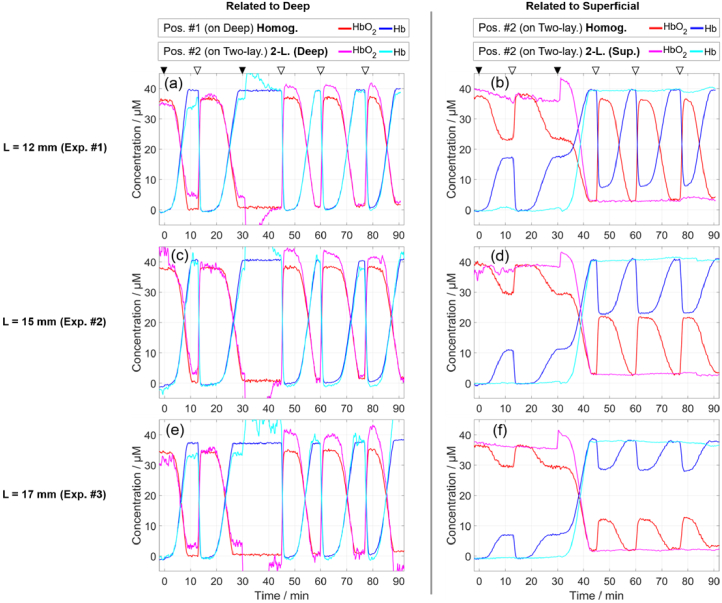
Concentrations of HbO_2_ and Hb in three experiments (three rows) involving blood. Curve-fitting method (Homog.) was used for measurements on Pos. #1 and on Pos. #2, and the latter was also analyzed with method based on moments (2-L. Deep and 2-L. Sup). For visualization, a moving average window (10 data points) was applied to all curves. Triangles at the top are explained in caption of Fig. 7.

**Fig. 12.**
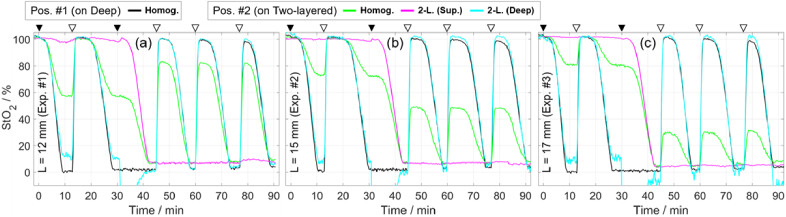
Determined StO_2_ in first (a), second (b), and third (c) experiments involving blood for two measurements and two methods of data analysis. The concentrations corresponding to the four StO_2_ curves are shown in Fig. 11. A moving average window (10 data points) was applied to all curves. Triangles at the top are explained in caption of Fig. 7.

**Fig. 13.**
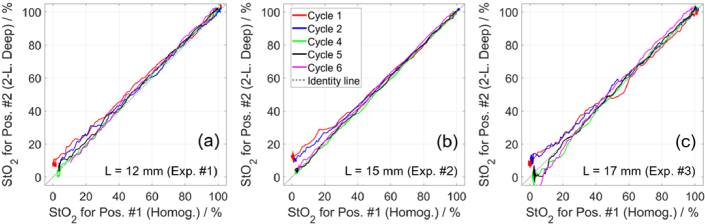
Comparison of StO_2_ time traces (from Fig. 12) for first (a), second (b), and third (c) experiments involving blood. Measurements on deep compartment were analyzed using curve-fitting method with homogeneous model (plotted along x-axis). Measurements on two-layered medium were analyzed using method based on moments with two-layered model (along y-axis).

In this section we present the results of the three experiments involving blood. The results are presented in the order according to the data analysis: the measured DTOFs and the calculated changes in moments (Fig. 7), the determined *µ*_a_ and *µ′*_s_ (Fig. 8 to 10), the determined concentrations of HbO_2_ and Hb (Fig. 11), and finally the determined StO_2_ (Fig. 12 and 13).

Figure 7 (a) shows DTOFs measured on Pos. #2, at different StO_2_ values in the two compartments, at 705 nm for which Δ*µ*_a_ is high. The Δ*µ*_a_ due to changes in StO_2_ depends on the difference of the molar absorption coefficients (shown in Fig. 8 (a)). For example, close to the isosbestic point, e.g. at 808 nm, Δ*µ*_a_ is close to zero. An increase in *µ*_a_ in any layer results in a narrower DTOF, i.e. the curve after the peak decreases at a faster rate. Sato et al. [90] used the slopes of the segments along the time axis to selectively determine the *µ*_a_ of different layers. The four DTOFs in Fig. 7 (a) support and demonstrate this concept. When only *µ*_a,Sup_ is increased (green curve), the slope of the curve at earlier time-channels changes, while the slope at the later time-channels (from about 4 ns) appears unchanged. When only *µ*_a,deep_ is increased (orange curve), the slope of the curve at later time-channels (from about 4 ns) is affected more compared to earlier time channels. The arrival time of photons is related to the mean penetration depth, and this leads to a relationship between *µ*_a_ at different depths and the slope of the DTOF at different times, as explained in [90]. When *µ*_a_ of both compartments is maximum (red curve), the curve has a small bump at about 4 ns (about 1 ns after the DTOF’s maximum). This bump is caused by the after-pulse peak in the IRF, which occurs about 1 ns after the IRF’s maximum and has a magnitude of about 1% of the IRF’s maximum, as shown in Fig. 5 (a).

Figure 7 (b, c) shows the time traces of changes in moments (Δ*m*_1_ and Δ*V*) relative to time zero, for measurements on Pos. #1 and Pos. #2, at 705 nm for which Δ*µ*_a_ is high. We found that the shape of the IRF did not vary between different experiments (on different days) and the IRF shifted by less than half of a time-channel (one time-channel was 12.22 ps). Therefore, we used the same time-channels for calculating moments for all three experiments, from 25% to 3% of the maximum as shown in Fig. 7 (a). The time trace of Δ*A* is not shown as it was not used in data analysis and it required correction for changes in the ND filter, which we changed to obtain good signals at high *µ*_a_ and avoid oversaturating detectors at low *µ*_a_.

The measurements on the deep compartment demonstrate the repeatability of the experiment, as they are independent of *L*. Noticeably, the rate of desaturation was slightly different in each experiment. For smaller *L*, the changes measured on the two-layered medium approach those measured on the deep compartment, as expected. By comparing the relative magnitudes of Δ*m*_1_ and Δ*V* for the two measurement positions, we can confirm that Δ*V* has a higher depth selectivity [48,88]. When both compartments were desaturated (e.g. at around 43 min), we expect the same changes in moments for measurements on both windows. The observed differences may be due to different *µ′*_s_ in the two compartments (shown in Fig. 10), which was caused by the addition of yeast as visible at 0 and 31 min in Fig. 7 (b, c) and Fig. 10. The values of *m*_1_ and *V* remained stable until the fourth deoxygenation cycle, after which they gradually decreased following a drift in *µ′*_s_ (Fig. 10).

Figure 8 shows the determined *µ*_a_ and *µ′*_s_ spectra for the deep compartment at maximum and minimum StO_2_, i.e. at first and second baselines, respectively, which are indicated in Fig. 7 (b). We chose two baseline regions, relative to which the changes in moments will later be calculated for determining Δ*µ*_a_, so that changes in StO_2_ occurred in only one compartment at a time. The first chosen baseline region is from 14.6 to 15.6 min, when StO_2_ was close to 100% in both compartments. The second region is from 58.8 to 59.8 min, which is after both compartments were fully deoxygenated. We used the second baseline for analyzing measurements after 31 min, which is after yeast was added to the superficial compartment. The *µ*_a_ spectra (Fig. 8 (a)) resemble the shapes of the molar absorption spectra (ε) of HbO_2_ and Hb. The differences are mainly due to the contribution of water’s *µ*_a_, which should be subtracted from the determined *µ*_a_ values for calculating concentrations. The *µ′*_s_ values (Fig. 8 (b)) decrease with wavelength, similar to the trend observed for Intralipid [81,84] and tissues [85,86]. The values of *µ*_a_ and *µ′*_s_ were well-repeatable for the three experiments.

Figure 9 shows the time traces of the determined *µ*_a_, which were obtained for two measurement positions and two methods of data analysis. The *µ*_a_ of the deep compartment (black curve) demonstrate five repeatable deoxygenation cycles, with no significant differences between the three experiments. However, after the fourth deoxygenation cycle (after 45 min), the *µ*_a_ slightly drifts upwards over time, as evidenced by the increasing minimum *µ*_a_ of different deoxygenation cycles, while *µ*′_s_ drifts downwards (Fig. 10). The maximum *µ*_a_ values (e.g. at around 30 min) differ for each experiment, with values of approximately 0.167, 0.173, and 0.158 cm^-1^ for Exp. #1, #2, and #3, respectively. The noise level expectedly increases for higher *µ*_a_ values, due to the lower count rate.

The comparison between the results obtained using the two methods (shown in Fig. 9) is consistent with the findings from the experiments involving ink (shown in Fig. 6). Analyzing the measurements on Pos. #2 using the curve-fitting method with a homogeneous model (green curves), produced *µ*_a_ values that are between the *µ*_a_ of the two compartments, and closer to the *µ*_a_ of the superficial compartment for larger *L*. The method based on moments accurately determined Δ*µ*_a,Sup_ and Δ*µ*_a,Deep_ (magenta and cyan curves) in all cycles except the third cycle (between 31 and 45 min). The accuracy of determined Δ*µ*_a,Deep_ (cyan curve) can be evaluated by comparing to the measurements on the deep compartment (black curve), and the accuracy of Δ*µ*_a,Sup_ (magenta curve) can be assessed based on the protocol, i.e. it was constant except during the third cycle. In the third cycle, the method does not account for Δ*µ*′_s,Sup_ caused by the yeast added at 31 min, which resulted in inaccurately determined Δ*µ*_a,Sup_ and especially Δ*µ*_a,Deep_. The method is insensitive to small changes in *µ′*_s,Deep_, but sensitive to *µ′*_s,Sup_, as demonstrated in Fig. 3 (b).

Figure 10 shows the time traces of the *µ*′_s_, which were determined together with *µ*_a_ (black and green curves in Fig. 9) using the curve-fitting method with a homogeneous model. The *µ*′_s_ measured on the deep compartment were stable up to the fourth cycle (45 min) and then began to decrease. This trend was observed for all wavelengths and in all three experiments. The trend is more similar between Exp. #1 and #3. Adding yeast at 0 min increased *µ*′_s_ by approximately 0.3 cm^-1^ in all three experiments.

The *µ*′_s_ values obtained from measurements on the two-layered medium (analyzed with a homogeneous model, which is suitable only when *µ*_a,Sup_ *= µ*_a,Deep_) are overestimated in the first three deoxygenation cycles (until 45 min, when *µ*_a,Sup_ ≤ *µ*_a,Deep_ as shown in Fig. 9) and then underestimated in the last three cycles (after 45 min, when *µ*_a,Sup_ ≥ *µ*_a,Deep_). This is a consequence of utilizing a homogeneous model for the analysis of measurements on a two-layered medium. Adding yeast at 31 min increased *µ*′_s_ by approximately 0.2 cm^-1^.

Figure 11 shows the time traces of the concentrations of HbO_2_ and Hb in the deep compartment (left column) and in the superficial compartment (right column), which were calculated using the determined *µ*_a_ at multiple wavelengths (shown in Fig. 9 for one wavelength). The results for measurements on Pos. #1, analyzed using the curve-fitting method with a homogeneous model (red and blue curves in the left panels), are consistent with the expected time traces of the concentrations based on the protocol used. In particular, during each deoxygenation cycle, all (or most) of HbO_2_ loses oxygen and becomes Hb (due to yeast consuming oxygen), while the total hemoglobin remains constant (traces of total hemoglobin are not shown). Then, bubbling oxygen results in all (or most) of Hb gaining oxygen and becoming HbO_2_ again. The total concentrations differ slightly for the three experiments, following the differences in the maximum of *µ*_a_ shown in Fig. 9. In the case of Pos. #2, analyzing the measurements using the curve-fitting method with a homogeneous model (red and blue curves in the right panels), provided concentrations of HbO_2_ and Hb that are between those of the two compartments and closer to the superficial compartment for larger *L*.

The concentrations obtained for the method based on moments with a two-layered model are consistent with the expected time traces based on the protocol. The concentrations for the deep compartment (magenta and cyan curves in the left panels) are similar to those obtained using measurements on Pos. #1 (red and blue curves). The concentrations for the superficial compartment (magenta and cyan curves in the right panels) are relatively constant during five cycles and change during the third cycle, as expected based on the protocol. The addition of yeast to the superficial compartment at 31 min had a clear influence on the determined concentrations. Negative values were obtained for HbO_2_ in the deep compartment when the determined *µ*_a,Deep_ had the highest error during the third cycle. Larger *L* lead to noisier results for the deep compartment and less noisy results for the superficial compartment.

The obtained results for HbO_2_ are noisier than for Hb for both data analysis methods. The reason is the higher values of ε of Hb compared to ε of HbO_2_ at most of the used wavelengths, visible in Fig. 8 (a). A change in Hb has a larger effect on *µ*_a_ than an equivalent change in HbO_2_.

Figure 12 shows the time traces of StO_2_, which were calculated using the concentrations in Fig. 11 and the formula StO_2_ *=* C_HbO2_ / (C_HbO2_ + C_Hb_), where C stands for concentration. Most StO_2_ values are between 0 and 100%, except in the third cycle where negative values of C_HbO2_ were obtained resulting in negative values of StO_2_. The StO_2_ in the deep compartment (black curve) follows five deoxygenation cycles from about 100% to 0%, as expected, but the maximum StO_2_ slightly reduces with each deoxygenation cycle in all three experiments. The StO_2_ values obtained from Pos. #2, analyzed with a homogeneous model (green curve), are between the StO_2_ values of the two compartments and approach the StO_2_ of superficial compartment for larger *L*.

The StO_2_ for the superficial compartment obtained from the two-layered measurements (magenta curve) is maximum for the first two cycles and minimum after the third cycle, which follows the protocol. The StO_2_ for the deep compartment (cyan curve) closely resembles the results of measurements on the deep compartment (black curve). However, the values deviate for some of the larger changes in StO_2_ relative to the baseline and more so for larger *L*.

The curves of StO_2_ in Fig. 12 resemble the curves of *µ*_a_ in Fig. 9, which is expected since StO_2_ calculation was based on these *µ*_a_ values along with the *µ*_a_ values at other wavelengths.

Figure 13 shows a comparison of the determined StO_2_ values for measurements on the deep compartment and on the two-layered medium. The StO_2_ curves (shown in Fig. 12) were smoothed using a moving average window (10 data points), which facilitated plotting curves in Fig. 13 rather than scattered data points. We compared the determined StO_2_ values during five deoxygenation cycles, between the maximum StO_2_ (after bubbling oxygen) and the minimum StO_2_ (plateau at the end of desaturation). The third cycle was excluded from this comparison because the StO_2_ in the deep compartment was constant throughout the cycle. This method of comparing simultaneous NIRS measurements was previously applied by Kleiser et al. [63,64] and in other studies [70]. The results show that the two methods produced agreeing values of StO_2_ throughout all cycles, as the curves are close to the identity line. However, in the first two cycles, the results obtained at low StO_2_ (i.e. furthest away from the baseline StO_2_ of approximately 100%) are noisiest and in part deviate from the identity line. In the last three cycles, the agreement is good at all StO_2_ values. The results for the deep compartment for measurements on the two-layered medium are noisier for larger *L* (as expected) and at lower StO_2_ (which corresponds to higher *µ*_a_ for most of the used wavelengths).

## 4. Discussion

This work has three main parts: (i) a new method for determining even large Δ*µ*_a_ in multiple layers, (ii) a new two-layered blood-lipid phantom, and (iii) the published MATLAB codes together with the measured TD-NIRS data for two experiments involving ink and three experiments involving blood.

### 4.1 New method

Previous implementations of analysis based on changes in moments for determining Δ*µ*_a_ were limited to a range of Δ*µ*_a_ within which the linearity assumption is valid. Therefore, they were suitable only for fNIRS applications, where small Δ*µ*_a_ are measured. Figures 2 (a) and 5 (b) show the changes in moments for a range of Δ*µ*_a_ values, demonstrating the progressive deviation from linearity as the magnitude of Δ*µ*_a_ increases. In this study, we have experimentally tested the proposed method’s capability to accurately determine also large Δ*µ*_a_ in two layers, i.e. in two compartments of the phantom. The method can now be applied also to NIRS applications that require obtaining absolute *µ*_a_, e.g. for determining StO_2_. In well-controlled experiments involving ink, we tested the method’s accuracy in determining 20 Δ*µ*_a_ steps (up to ± 0.10 cm^−1^) in the deep or the superficial compartment. The experiments involving blood demonstrated the ability to determine StO_2_ in two layers during dynamic StO_2_ changes. For methods that are capable of determining only small Δ*µ*_a_, the baseline optical properties had to be updated after a Δ*µ*_a_ step to satisfy the linearity assumption for determining the next Δ*µ*_a_ [43]. However, the new method removes the restriction on the range of Δ*µ*_a_ values that can be accurately determined. Another advantageous feature of the method is its low sensitivity to the selected range of time channels used in the calculations. This is a consequence of using the same range for computing the moments of both the measured DTOF and the simulated DTOF convolved with IRF. In contrast, other methods that utilize moments [75] or changes in moments [41] suffer from errors caused by the limited integration range. These errors are sensitive to the selected range of time channels, as explained by Jelzow et al. [43].

The proposed method requires knowing the baseline optical properties, which we obtained by measuring on each compartment of the phantom separately and using the curve-fitting method with the homogeneous model. However, for analyzing *in-vivo* measurements, it remains a challenge to develop an accurate and robust approach for estimating the baseline optical properties of two layers as well as thickness *L*, e.g. [28,33–36]. It can be particularly challenging to estimate *L* using a single DTOF, e.g. if the optical properties of both layers are the same, then any value of *L* will fit the data, i.e. the DTOF. We demonstrated in Fig. 2 (a) that for obtained changes in two moments, there is a unique solution of Δ*µ*_a_ in two layers. In fNIRS applications (small Δ*µ*_a_), it is sufficient to know approximate values of the baseline parameters (supported by Fig. 3). This is consistent with the established method based on moments, which requires estimates of the values of all baseline parameters for generating sensitivity factors [41]. However, the estimation of *L* and baseline optical properties is an important and needed topic for further research.

The accuracy of generating the theoretical DTOFs governs the accuracy of the recovered optical properties. One way to enhance the accuracy of theoretical DTOFs is by using Monte Carlo simulations, which is considered as the gold-standard method for simulating NIRS data [30]. The accuracy can be further improved by using a more realistic geometrical model, e.g. an anatomical head model from MRI data [91].

Sassaroli et al. [92] derived formulas for light propagation to obtain moments directly, eliminating the need to generate DTOFs and thereby reducing computational time. This approach can be particularly advantageous when incorporating changes in moments at multiple source-detector distances, as the computational time for the LMA may grow exponentially with the number of source-detector distances used.

The current implementation of the method based on moments uses only two DTOFs (at baseline and after Δ*µ*_a_). To further enhance the method, more DTOFs could be incorporated in data analysis. For example, Jelzow et al. [43] fitted a third-order polynomial to the measured changes in moments before using them to determine Δ*µ*_a_, which can improve the signal-to-noise ratio. Yang et al. [74,76] proposed a new approach that exploits measurements at multiple source-detector distances, and demonstrated the benefits of incorporating spatial information in addition to time-domain measurements, which improved reliability and reduced uncertainty in determining the absolute optical properties of multiple layers. While the present study used measurements at a single source-detector distance, it is important to note that the proposed method can be extended to use changes in moments at multiple source-detector distances.

We did not use the intensity information (Δ*A*) for determining Δ*µ*_a_ because it required careful data processing to account for potential drifts and changes in the output of the laser. Incorporating Δ*A* information could greatly improve data analysis. We weighted changes in moments by their uncertainty due to photon noise (Poisson statistics) in Eq. (3), to account for the different units and varying levels of uncertainty. A more accurate estimate of the uncertainty could incorporate also the instrumental noise for each moment, which can be measured once for a system [43].

We did not report the uncertainties of the determined optical properties in this study, as it is dependent on several factors including the selected laser power and the acquisition time. The uncertainty of the fitted parameters (for the LMA method) due to photon statistics can be obtained using the method described in [93].

When calculating concentrations of oxy- and deoxyhemoglobin, we used the determined *µ*_a_ values at multiple wavelengths, but without accounting for the differences in their uncertainties. The determined *µ*_a_ at different wavelengths have varying levels of uncertainty, which relates to the *µ*_a_ spectrum of the medium (higher uncertainty for higher *µ*_a_) and to the system used (e.g. if different wavelengths have different power or detection sensitivity). Future studies should aim to incorporate information about uncertainties to improve the determination of hemoglobin concentrations and hence StO_2_, e.g. as in [35].

### 4.2 Measured data and MATLAB codes

The measured TD-NIRS data and the MATLAB codes used in this study were made publicly available [68]. The data is suitable for testing new data analysis methods for tissue oximeters.

The LMA is a search-based algorithm that allows entering the values of known parameters and then determining the unknown parameters. We implemented two LMA algorithms, both of which have the feature of allowing the user to choose which parameters to determine. The first algorithm uses the DTOF as an input and determines any of the eight parameters: *µ*_a_, *µ′*_s_, and/or *L* (layer’s thickness), for up to three layers (the deepest layer is assumed semi-infinite). We used it to determine *µ*_a_ and *µ′*_s_ assuming a homogeneous medium. The second algorithm uses changes in moments as an input and determines any of the following: Δ*µ*_a_, Δ*µ′*_s_, and/or *L*, for up to three layers. We used it to determine Δ*µ*_a,Sup_ and Δ*µ*_a,Deep_ of a two-layered medium. Future studies may be interested in using this code to determine Δ*µ*_a_ in two layers (superficial and deep) of a three-layered medium, with the assumption that the *µ*_a_ of the middle layer is constant (mimicking the skull). Additionally, the codes can be modified to use DTOFs (in the first algorithm) or changes in moments (in the second algorithm) at multiple source-detector distances.

### 4.3 Phantom

The phantom allows repeatedly deoxygenating and oxygenating blood in two compartments individually (the results for oxygenating the superficial compartment were not presented), and carrying out simultaneous NIRS measurements on any of the compartments: the two-layered medium (variable superficial layer thickness), the deep (semi-infinite medium), and/or the superficial (semi-infinite medium). The measurements on individual compartments can be analyzed employing a homogeneous model, providing a reference for the optical properties within each compartment, as was done in this study (Fig. 6). The repeatability within and between experiments was demonstrated by carrying out six deoxygenation cycles in three experiments (18 cycles in total). The constructed phantom enables carrying out reproducible experiments, and has good durability and ease of use. The hemoglobin concentrations and the corresponding StO_2_ levels in each compartment followed the expected desaturation trends (StO_2_ decreased from 100% to 0%) during all deoxygenation cycles.

The rate of desaturation varied slightly across the three experiments, as seen from the changes in moments in Fig. 7. One potential cause could be a slight difference in the amount of yeast that was added to the phantom. After pouring the mixture containing yeast into the phantom, a small amount of yeast may have adhered to the walls of the beaker, leading to an uncertainty in the amount of yeast added. The temperature of the phantom affects the rate of desaturation, but the temperature was stable as measured with two thermometers.

We found that adding yeast to the solution resulted in an increase in *µ′*_s_ (Fig. 10). After the start of the fourth cycle (after 45 min), the *µ*′_s_ in the deep compartment began to steadily decrease (visible in Fig. 7 and 10). This decrease had more similar trends in the first and third experiments, in which the same blood bag was used. The decrease in *µ*′_s,Deep_ may be related to either the yeast, which was mixing for over 45 minutes, or the blood, which was kept in a desaturated state for an extended period of time (during the third cycle). Ostojic et al. [94] studied the gradual decrease of *µ*′_s_ over time in phantoms with blood and proposed possible explanations.

The large size of the phantom requires large amounts of ingredients, which increases the cost of conducting experiments, but more importantly, it makes the phantom easier to control. Due to the large volume, the temperature of the phantom changes at a slow rate, making it easier to maintain it at approximately 37 °C. Additionally, the experiments can be carried out with higher accuracy when handling larger amounts of ingredients, improving the reproducibility of experiments. For example, we added about 3.5 g of Mixture #2 for each step of Δ*µ*_a_, whereas in another two-layered phantom, the authors needed to add only about 0.7 g to achieve similar Δ*µ*_a_ [43].

The measurement windows and/or the separating window of the phantom (shown in Fig. 1) could be covered with a layer of material that has tissue-mimicking optical properties for mimicking the skin and/or the skull. This could be achieved using the silicone-based layers presented by Kleiser et al. [63,64] and/or the solid layers presented by Izzetoglu et al. [58], which can be created with variable tissue-mimicking optical properties and thicknesses.

One of the limitations of the study is the absence of an independent reference measurement of StO_2_, which limits the assessment of the accuracy of the determined StO_2_ values since the true values are unknown. In this study, we compared two measurements of StO_2_ (on the deep compartment and on the two-layered medium), both of which were measured by the same system. Kleiser et al. [63,64] showed that different NIRS tissue oximeters can produce different StO_2_ values, even when they simultaneously measure on the same phantom. Furthermore, the StO_2_ values also depend on the used method of data analysis. The partial pressure of oxygen (pO_2_) was proposed as a reference measure in previous studies, but it has major challenges [63,67]. Obtaining reliable reference values of StO_2_ would be a significant advancement in the assessment of accuracy, and for calibrating different systems and methods of data analysis.

## 5. Conclusions

We introduced and experimentally tested a new method for accurately determining even large Δ*µ*_a_ in multiple layers, which utilizes (i) changes in moments (making use of two DTOFs, at baseline and after Δ*µ*_a_ in two layers, and the knowledge of the optical properties at baseline), (ii) an analytical solution of the diffusion equation for a multi-layered medium, and (iii) the Levenberg–Marquardt algorithm. We applied the method for tissue oximetry for determining StO_2_ in two layers (the absolute *µ*_a_ was obtained by summing the baseline *µ*_a_ and the determined Δ*µ*_a_).

We used the established concept of a blood-lipid phantom and constructed a phantom with two such compartments (mimicking superficial and deep layers, with a variable superficial layer thickness), allowing for the first time, independent control of StO_2_ of blood in two compartments. The phantom may be used for performance assessment, such as testing NIRS tissue oximeters or new methods of data analysis, as well as for studying the influence of the superficial compartment on the estimation of StO_2_ in the deep compartment.

The results of five experiments, two of which varied the concentration of ink and three of which varied the StO_2_ of blood, demonstrated a precise control of the phantom and a high reproducibility of deoxygenation cycles within and between experiments. Also, the accurate determination of *µ*_a_ and/or StO_2_ in both compartments, using time-domain NIRS data measured on two-layered medium, was confirmed experimentally.

## Data Availability

Data and MATLAB codes used in this study were made available under the GNU General Public License v3.0 [68].

## References

[1] Wong Z. Z. Chiong X. H. Chaw S. H. Hashim N. H. B. M. Abidin M. F. B. Z. Yunus S. N. B. Subramaniam T. Ng K. T., “The Use of Cerebral Oximetry in Surgery: A Systematic Review and Meta-analysis of Randomized Controlled Trials,” J. Cardiothorac. Vasc. Anesth. 36(7), 2002–2011 (2022). 10.1053/j.jvca.2021.09.046 34657798

[2] Sanfilippo F. Murabito P. Messina A. Dezio V. Busalacchi D. Ristagno G. Cecconi M. Astuto M., “Cerebral regional oxygen saturation during cardiopulmonary resuscitation and return of spontaneous circulation: A systematic review and meta-analysis,” Resuscitation 159, 19–27 (2021). 10.1016/j.resuscitation.2020.12.002 33333181

[3] Viderman D. Abdildin Y. G., “Near-Infrared Spectroscopy in Neurocritical Care: A Review of Recent Updates,” World Neurosurgery 151, 23–28 (2021). 10.1016/j.wneu.2021.04.054 33895369

[4] Roldán M. Abay T. Y. Kyriacou P. A., “Non-Invasive Techniques for Multimodal Monitoring in Traumatic Brain Injury: Systematic Review and Meta-Analysis,” Journal of neurotrauma 37(23), 2445–2453 (2020). 10.1089/neu.2020.7266 32821023

[5] Chen W.-L. Wagner J. Heugel N. Sugar J. Lee Y.-W. Conant L. Malloy M. Heffernan J. Quirk B. Zinos A. Beardsley S. A. Prost R. Whelan H. T., “Functional Near-Infrared Spectroscopy and Its Clinical Application in the Field of Neuroscience: Advances and Future Directions,” Front. Neurosci. 14, 724 (2020). 10.3389/fnins.2020.00724 32742257 PMC7364176

[6] Chen Y. Shen Z. Shao Z. Yu P. Wu J., “Free Flap Monitoring Using Near-Infrared Spectroscopy: A Systemic Review,” Ann. Plast. Surg. 76(5), 590–597 (2016). 10.1097/SAP.0000000000000430 25664408

[7] Sørensen H., “Near infrared spectroscopy evaluated cerebral oxygenation during anesthesia,” Danish Medical Journal 63(12), B5318 (2016), PhD Thesis. 27910802

[8] Green M. S. Sehgal S. Tariq R., “Near-Infrared Spectroscopy: The New Must Have Tool in the Intensive Care Unit?” Semin Cardiothorac Vasc Anesth 20(3), 213–224 (2016). 10.1177/1089253216644346 27206637

[9] Boezeman R. P. Moll F. L. Ünlü Ç. de Vries J. P., “Systematic review of clinical applications of monitoring muscle tissue oxygenation with near-infrared spectroscopy in vascular disease,” Microvasc. Res. 104, 11–22 (2016). 10.1016/j.mvr.2015.11.004 26576829

[10] Bale G. Mitra S. Tachtsidis I., “Metabolic brain measurements in the newborn: Advances in optical technologies,” Physiol. Rep. 8(17), e1458 (2020). 10.14814/phy2.14548 PMC7507543 32889790

[11] Lange F. Giannoni L. Tachtsidis I., “The Use of Supercontinuum Laser Sources in Biomedical Diffuse Optics: Unlocking the Power of Multispectral Imaging,” Appl. Sci. 11(10), 4616 (2021). 10.3390/app11104616

[12] Ban H. Barrett G. Borisevich A. et al., “Kernel flow: a high channel count scalable time-domain functional near-infrared spectroscopy system,” J. Biomed. Opt. 27, 074710 (2022). 10.1117/1.jbo.27.7.074710 35043610 PMC8765296

[13] Harvey-Jones K. Lange F. Tachtsidis I. Robertson N. J. Mitra S., “Role of optical neuromonitoring in neonatal encephalopathy—current state and recent advances,” Front. Pediatr. 9, 1 (2021). 10.3389/fped.2021.653676 PMC8062863 33898363

[14] Althobaiti M. Al-Naib I., “Recent Developments in Instrumentation of Functional Near-Infrared Spectroscopy Systems,” Appl. Sci. 10(18), 6522 (2020). 10.3390/app10186522

[15] Konugolu Venkata Sekar S. Lanka P. Farina A. Dalla Mora A. Andersson-Engels S. Taroni P. Pifferi A., “Broadband Time Domain Diffuse Optical Reflectance Spectroscopy: A Review of Systems, Methods, and Applications,” Appl. Sci. 9(24), 5465 (2019). 10.3390/app9245465

[16] Lange F. Tachtsidis I., “Clinical Brain Monitoring with Time Domain NIRS: A Review and Future Perspectives,” Appl. Sci. 9(8), 1612 (2019). 10.3390/app9081612

[17] Bale G. Elwell C. Tachtsidis I., “From Jöbsis to the present day: a review of clinical near-infrared spectroscopy measurements of cerebral cytochrome-c-oxidase,” J. Biomed. Opt. 21(9), 091307 (2016). 10.1117/1.JBO.21.9.091307 27170072

[18] Tachtsidis I. Scholkmann F., “False positives and false negatives in functional near-infrared spectroscopy: issues, challenges, and the way forward,” Neurophotonics 3(3), 031405 (2016). 10.1117/1.NPh.3.3.031405 27054143 PMC4791590

[19] Giles B. Cristianne F. Angelo S. Sergio F., “Dual-slope imaging of cerebral hemodynamics with frequency-domain near-infrared spectroscopy,” Neurophotonics 10(01), 013508 (2023). 10.1117/1.NPh.10.1.013508 36601543 PMC9807277

[20] Farrell T. J. Patterson M. S. Essenpreis M., “Influence of layered tissue architecture on estimates of tissue optical properties obtained from spatially resolved diffuse reflectometry,” Appl. Opt. 37(10), 1958–1972 (1998). 10.1364/AO.37.001958 18273116

[21] Davie Sophie N. Grocott Hilary P., “Impact of Extracranial Contamination on Regional Cerebral Oxygen Saturation: A Comparison of Three Cerebral Oximetry Technologies,” Anesthesiology 116(4), 834–840 (2012). 10.1097/ALN.0b013e31824c00d7 22343469

[22] Nasseri N. Kleiser S. Ostojic D. Karen T. Wolf M., “Quantifying the effect of adipose tissue in muscle oximetry by near infrared spectroscopy,” Biomed. Opt. Express 7(11), 4605–4619 (2016). 10.1364/BOE.7.004605 27895999 PMC5119599

[23] Fabrizio Martelli T. B. Del Bianco Samuele Liemert André Kienle Alwin, *Light Propagation through Biological Tissue and Other Diffusive Media: Theory, Solutions, and Validations* , Second Edition (SPIE, 2022).

[24] Patterson M. S. Chance B. Wilson B. C., “Time resolved reflectance and transmittance for the noninvasive measurement of tissue optical properties,” Appl. Opt. 28(12), 2331–2336 (1989). 10.1364/AO.28.002331 20555520

[25] Haskell R. C. Svaasand L. O. Tsay T.-T. Feng T.-C. McAdams M. S. Tromberg B. J., “Boundary conditions for the diffusion equation in radiative transfer,” J. Opt. Soc. Am. A 11(10), 2727–2741 (1994). 10.1364/JOSAA.11.002727 7931757

[26] Arridge S. R. Cope M. Delpy D. T., “The theoretical basis for the determination of optical pathlengths in tissue: temporal and frequency analysis,” Phys. Med. Biol. 37(7), 1531–1560 (1992). 10.1088/0031-9155/37/7/005 1631197

[27] Helton M. Zerafa S. Vishwanath K. Mycek M.-A., “Efficient computation of the steady-state and time-domain solutions of the photon diffusion equation in layered turbid media,” Sci. Rep. 12, 18979 (2022). 10.1038/s41598-022-22649-4 . 36347893 PMC9643457

[28] Geiger S. Reitzle D. Liemert A. Kienle A., “Determination of the optical properties of three-layered turbid media in the time domain using the P3 approximation,” OSA Continuum 2(6), 1889–1899 (2019). 10.1364/OSAC.2.001889

[29] Dehghani H. Eames M. E. Yalavarthy P. K. Davis S. C. Srinivasan S. Carpenter C. M. Pogue B. W. Paulsen K. D., “Near infrared optical tomography using NIRFAST: Algorithm for numerical model and image reconstruction,” Commun Numer Methods Eng 25, 711–732 (2008). 10.1002/cnm.1162 20182646 PMC2826796

[30] Wojtkiewicz S. Liebert A., “Parallel, multi-purpose Monte Carlo code for simulation of light propagation in segmented tissues,” Biocybernetics and Biomedical Engineering 41(4), 1303–1321 (2021). 10.1016/j.bbe.2021.03.001

[31] Liebert A. Wabnitz H. Zolek N. Macdonald R., “Monte Carlo algorithm for efficient simulation of time-resolved fluorescence in layered turbid media,” Opt. Express 16(17), 13188–13202 (2008). 10.1364/OE.16.013188 18711557

[32] Liemert A. Kienle A., “Application of the Laplace transform in time-domain optical spectroscopy and imaging,” J. Biomed. Opt. 20(11), 110502 (2015). 10.1117/1.JBO.20.11.110502 26580698

[33] Fabrizio M. Samuele Del B. Lorenzo S. Stefano C. Paola Di N. Tiziano B. Alexander J. Rainer M. Heidrun W., “Optimal estimation reconstruction of the optical properties of a two-layered tissue phantom from time-resolved single-distance measurements,” J. Biomed. Opt. 20(11), 115001 (2015). 10.1117/1.JBO.20.11.115001 26524677

[34] García H. A. Iriarte D. I. Pomarico J. A. Grosenick D. Macdonald R., “Retrieval of the optical properties of a semiinfinite compartment in a layered scattering medium by single-distance, time-resolved diffuse reflectance measurements,” J. Quant. Spectrosc. Radiat. Transfer 189, 66–74 (2017). 10.1016/j.jqsrt.2016.11.018

[35] García H. Baez G. Pomarico J., “Simultaneous retrieval of optical and geometrical parameters of multilayered turbid media via state-estimation algorithms,” Biomed. Opt. Express 9(8), 3953–3973 (2018). 10.1364/BOE.9.003953 30338167 PMC6191609

[36] Baez G. R. García H. Grosenick D. Wabnitz H., “Implementation of the extended Kalman filter for determining the optical and geometrical properties of turbid layered media by time-resolved single distance measurements,” Biomed. Opt. Express 11(1), 251–266 (2020). 10.1364/BOE.11.000251 32010514 PMC6968768

[37] Mosca S. Lanka P. Stone N. Konugolu Venkata Sekar S. Matousek P. Valentini G. Pifferi A., “Optical characterisation of porcine tissues from various organs in the 650–1100 nm range using time-domain diffuse spectroscopy,” Biomed. Opt. Express 11(3), 1697–1706 (2020). 10.1364/BOE.386349 32206436 PMC7075607

[38] Konugolu Venkata Sekar S. Farina A. Dalla Mora A. Lindner C. Pagliazzi M. Mora M. Aranda G. Dehghani H. Durduran T. Taroni P. Pifferi A., “Broadband (550–1350 nm) diffuse optical characterization of thyroid chromophores,” Sci. Rep. 8(1), 10015 (2018). 10.1038/s41598-018-27684-8 29968735 PMC6030074

[39] Hallacoglu B. Sassaroli A. Fantini S., “Optical characterization of two-layered turbid media for non-invasive, absolute oximetry in cerebral and extracerebral tissue,” PLoS One 8(5), e64095 (2013). 10.1371/journal.pone.0064095 23724023 PMC3660388

[40] Steinbrink J. Wabnitz H. Obrig H. Villringer A. Rinneberg H., “Determining changes in NIR absorption using a layered model of the human head,” Phys. Med. Biol. 46(3), 879–896 (2001). 10.1088/0031-9155/46/3/320 11277232

[41] Liebert A. Wabnitz H. Steinbrink J. Obrig H. Möller M. Macdonald R. Villringer A. Rinneberg H., “Time-Resolved Multidistance Near-Infrared Spectroscopy of the Adult Head: Intracerebral and Extracerebral Absorption Changes from Moments of Distribution of Times of Flight of Photons,” Appl. Opt. 43(15), 3037–3047 (2004). 10.1364/AO.43.003037 15176190

[42] Liebert A. Wabnitz H. Elster C., “Determination of absorption changes from moments of distributions of times of flight of photons: optimization of measurement conditions for a two-layered tissue model,” J. Biomed. Opt. 17(5), 057005 (2012). 10.1117/1.JBO.17.5.057005 22612144

[43] Jelzow A. Wabnitz H. Tachtsidis I. Kirilina E. Brühl R. Macdonald R., “Separation of superficial and cerebral hemodynamics using a single distance time-domain NIRS measurement,” Biomed. Opt. Express 5(5), 1465–1482 (2014). 10.1364/BOE.5.001465 24877009 PMC4026903

[44] Milej D. Abdalmalak A. McLachlan P. Diop M. Liebert A. St Lawrence K., “Subtraction-based approach for enhancing the depth sensitivity of time-resolved NIRS,” Biomed. Opt. Express 7(11), 4514–4526 (2016). 10.1364/BOE.7.004514 27895992 PMC5119592

[45] Kacprzak M. Liebert A. Staszkiewicz W. Gabrusiewicz A. Sawosz P. Madycki G. Maniewski R., “Application of a time-resolved optical brain imager for monitoring cerebral oxygenation during carotid surgery,” J. Biomed. Opt. 17(1), 016002 (2012). 10.1117/1.JBO.17.1.016002 22352652

[46] Jelzow A., “In vivo quantification of absorption changes in the human brain by time-domain diffuse near-infrared spectroscopy,” PhD Thesis (2013).

[47] Antonio O.-M. De’Ja R. Jessica A. Parya F. Yuanyuan G. Bernhard Z. Meryem A. Y. David A. B., “How much do time-domain functional near-infrared spectroscopy (fNIRS) moments improve estimation of brain activity over traditional fNIRS?” Neurophotonics 10(01), 013504 (2022). 10.1117/1.NPh.10.1.013504 36284602 PMC9587749

[48] Wabnitz H. Contini D. Spinelli L. Torricelli A. Liebert A., “Depth-selective analysis in time-domain optical brain imaging: moments vs time windows,” Biomed. Opt. Express 11(8), 4224–4243 (2020). 10.1364/BOE.396585 32923038 PMC7449728

[49] Sudakou A. Wabnitz H. Yang L. Contini D. Spinelli L. Torricelli A. Liebert A., *Depth selectivity in time-domain fNIRS by analyzing moments and time windows* (SPIE, 2021).

[50] Gerega A. Milej D. Weigl W. Kacprzak M. Liebert A., “Multiwavelength time-resolved near-infrared spectroscopy of the adult head: Assessment of intracerebral and extracerebral absorption changes,” Biomed. Opt. Express 9(7), 2974 (2018). 10.1364/BOE.9.002974 29984079 PMC6033559

[51] Hornberger C. Wabnitz H., “Approaches for calibration and validation of near-infrared optical methods for oxygenation monitoring,” Biomed. Tech. 63(5), 537–546 (2018). 10.1515/bmt-2017-0116 29425103

[52] Lanka P. Yang L. Orive-Miguel D. et al., “Multi-laboratory performance assessment of diffuse optics instruments: the BitMap exercise,” J. Biomed. Opt. 27(07), 074716 (2022). 10.1117/1.JBO.27.7.074716 35701869 PMC9199954

[53] Wabnitz H. Taubert D. Mazurenka M. et al., “Performance assessment of time-domain optical brain imagers, part 1: basic instrumental performance protocol,” J. Biomed. Opt. 19(8), 086010 (2014). 10.1117/1.JBO.19.8.086010 25121479

[54] Wabnitz H. Jelzow A. Mazurenka M. et al., “Performance assessment of time-domain optical brain imagers, part 2: nEUROPt protocol,” J. Biomed. Opt. 19(8), 086012 (2014). 10.1117/1.JBO.19.8.086012 25121480

[55] Pifferi A. Torricelli A. Bassi A. Taroni P. Wabnitz H. Grosenick D. Möller M. Macdonald R. Swartling J. Svensson T. Andersson-Engels S. Veen R. Sterenborg H. Tualle J.-M. Halien N. Avrillier S. Whelan M. Stamm H., “Performance assessment of photon migration instruments: The MEDPHOT protocol,” Appl. Opt. 44(11), 2104–2114 (2005). 10.1364/AO.44.002104 15838951

[56] Yücel M. A. Lühmann A. V. Scholkmann F. et al., “Best practices for fNIRS publications,” Neurophotonics 8(01), 012101 (2021). 10.1117/1.NPh.8.1.012101 33442557 PMC7793571

[57] Hwang J. Ramella-Roman J. C. Nordstrom R., “Introduction: Feature Issue on Phantoms for the Performance Evaluation and Validation of Optical Medical Imaging Devices,” Biomed. Opt. Express 3(6), 1399–1403 (2012). 10.1364/BOE.3.001399 22741084 PMC3370978

[58] Izzetoglu M. Du J. Izzetoglu K. Ayaz H. Onaral B. Dor B. B., “Multilayer, dynamic, mixed solid/liquid human head models for the evaluation of near infrared spectroscopy systems,” IEEE Trans. Instrum. Meas. 69(10), 8441–8451 (2020). 10.1109/TIM.2020.2990261

[59] Afshari A. Ghassemi P. Lin J. Halprin M. Wang J. Mendoza G. Weininger S. Pfefer T. J., “Cerebral oximetry performance testing with a 3D-printed vascular array phantom,” Biomed. Opt. Express 10(8), 3731–3746 (2019). 10.1364/BOE.10.003731 31452971 PMC6701524

[60] Sekar S. K. V. Pacheco A. Martella P. Li H. Lanka P. Pifferi A. Andersson-Engels S., “Solid phantom recipe for diffuse optics in biophotonics applications: a step towards anatomically correct 3D tissue phantoms,” Biomed. Opt. Express 10(4), 2090–2100 (2019). 10.1364/BOE.10.002090 31061772 PMC6484985

[61] Pacheco A. Li H. Chakravarty M. Sekar S. Andersson-Engels S., “Anthropomorphic optical phantom of the neonatal thorax: a key tool for pulmonary studies in preterm infants,” J. Biomed. Opt. 25, 115001 (2020). 10.1117/1.JBO.25.11.115001 33205636 PMC7670093

[62] Liu G. Huang K. Jia Q. Liu S. Shen S. Li J. Dong E. Lemaillet P. Allen D. W. Xu R. X., “Fabrication of a multilayer tissue-mimicking phantom with tunable optical properties to simulate vascular oxygenation and perfusion for optical imaging technology,” Appl. Opt. 57(23), 6772–6780 (2018). 10.1364/AO.57.006772 30129625

[63] Kleiser S. Nasseri N. Andresen B. Greisen G. Wolf M., “Comparison of tissue oximeters on a liquid phantom with adjustable optical properties,” Biomed. Opt. Express 7(8), 2973–2992 (2016). 10.1364/BOE.7.002973 27570691 PMC4986807

[64] Kleiser S. Ostojic D. Andresen B. Nasseri N. Isler H. Scholkmann F. Karen T. Greisen G. Wolf M., “Comparison of tissue oximeters on a liquid phantom with adjustable optical properties: an extension,” Biomed. Opt. Express 9(1), 86–101 (2018). 10.1364/BOE.9.000086 29359089 PMC5772591

[65] Lange F. Dunne L. Hale L. Tachtsidis I., “MAESTROS: A Multiwavelength Time-Domain NIRS System to Monitor Changes in Oxygenation and Oxidation State of Cytochrome-C-Oxidase,” IEEE J. Sel. Top. Quantum Electron. 25(1), 1–12 (2019). 10.1109/JSTQE.2018.2833205 PMC6054019 30450021

[66] Izzetoglu M. Pourrezaei K. Du J. Shewokis P. A., “Evaluation of cerebral tissue oximeters using multilayered dynamic head models,” IEEE Trans. Instrum. Meas. 70, 1–12 (2021). 10.1109/TIM.2021.306646933776080

[67] Rejmstad P. Johansson J. D. Haj-Hosseini N. Wårdell K., “A method for monitoring of oxygen saturation changes in brain tissue using diffuse reflectance spectroscopy,” J. Biophotonics 10(3), 446–455 (2017). 10.1002/jbio.201500334 27094015

[68] Sudakou A. Wabnitz H. Liemert A. Wolf M. Liebert A., “TD-NIRS Data and MATLAB Codes used for this study,” Github 2023, https://github.com/asudakou/Analyzing_TD-NIRS.

[69] Gerega A. Milej D. Weigl W. Botwicz M. Zolek N. Kacprzak M. Wierzejski W. Toczylowska B. Mayzner-Zawadzka E. Maniewski R. Liebert A., “Multiwavelength time-resolved detection of fluorescence during the inflow of indocyanine green into the adult's brain,” J. Biomed. Opt. 17, 087001 (2012). 10.1117/1.jbo.17.8.087001 23224200

[70] Sudakou A. Lange F. Isler H. Lanka P. Wojtkiewicz S. Sawosz P. Ostojic D. Wolf M. Pifferi A. Tachtsidis I. Liebert A. Gerega A., “Time-domain NIRS system based on supercontinuum light source and multi-wavelength detection: validation for tissue oxygenation studies,” Biomed. Opt. Express 12(10), 6629–6650 (2021). 10.1364/BOE.431301 34745761 PMC8548017

[71] Ntziachristos V. Chance B., “Accuracy limits in the determination of absolute optical properties using time-resolved NIR spectroscopy,” Med. Phys. 28(6), 1115–1124 (2001). 10.1118/1.1373674 11439481

[72] Liebert A. Wabnitz H. Grosenick D. Macdonald R., “Fiber dispersion in time domain measurements compromising the accuracy of determination of optical properties of strongly scattering media,” J. Biomed. Opt. 8(3), 512 (2003). 10.1117/1.1578088 12880358

[73] Pirovano I. Re R. Candeo A. Contini D. Torricelli A. Spinelli L., “Instrument response function acquisition in reflectance geometry for time-resolved diffuse optical measurements,” Biomed. Opt. Express 11(1), 240–250 (2020). 10.1364/BOE.380996 32010513 PMC6968769

[74] Yang L. Wabnitz H. Gladytz T. Macdonald R. Grosenick D., “Spatially-enhanced time-domain NIRS for accurate determination of tissue optical properties,” Opt. Express 27(19), 26415 (2019). 10.1364/OE.27.026415 31674524

[75] Liebert A. Wabnitz H. Grosenick D. Moller M. Macdonald R. Rinneberg H., “Evaluation of optical properties of highly scattering media by moments of distributions of times of flight of photons,” Appl. Opt. 42(28), 5785–5792 (2003). 10.1364/AO.42.005785 14528944

[76] Yang L. Wabnitz H. Gladytz T. Sudakou A. Macdonald R. Grosenick D., “Space-enhanced time-domain diffuse optics for determination of tissue optical properties in two-layered structures,” Biomed. Opt. Express 11(11), 6570–6589 (2020). 10.1364/BOE.402181 33282509 PMC7687957

[77] Helton M. Mycek M.-A. Vishwanath K., “Reconstruction of optical coefficients in turbid media using time-resolved reflectance and calibration-free instrument response functions,” Biomed. Opt. Express 13(3), 1595–1608 (2022). 10.1364/BOE.447685 35414997 PMC8973157

[78] Kollias N. Gratzer W. B., “Tabulated molar extinction coefficient for hemoglobin in water,” Wellman Laboratories, Harvard Medical School, Boston 5, 150–161 (1999).

[79] Matcher S. J. Cope M. Delpy D. T., “Use of the water absorption spectrum to quantify tissue chromophore concentration changes in near-infrared spectroscopy,” Phys. Med. Biol. 39(1), 177–196 (1994). 10.1088/0031-9155/39/1/011 7651995

[80] Bianco S. D. Martelli F. Cignini F. Zaccanti G. Pifferi A. Torricelli A. Bassi A. Taroni P. Cubeddu R., “Liquid phantom for investigating light propagation through layered diffusive media,” Opt. Express 12(10), 2102–2111 (2004). 10.1364/OPEX.12.002102 19475045

[81] Ninni P. D. Martelli F. Zaccanti G., “Intralipid: towards a diffusive reference standard for optical tissue phantoms,” Phys. Med. Biol. 56(2), N21–28 (2011). 10.1088/0031-9155/56/2/N01 21160111

[82] Ninni P. D. Martelli F. Zaccanti G., “The use of India ink in tissue-simulating phantoms,” Opt. Express 18(26), 26854–26865 (2010). 10.1364/OE.18.026854 21196962

[83] Spinelli L. Martelli F. Farina A. Pifferi A. Torricelli A. Cubeddu R. Zaccanti G., “Calibration of scattering and absorption properties of a liquid diffusive medium at NIR wavelengths. Time-resolved method,” Opt. Express 15(11), 6589–6604 (2007). 10.1364/OE.15.006589 19546968

[84] Spinelli L. Botwicz M. Zolek N. et al., “Determination of reference values for optical properties of liquid phantoms based on Intralipid and India ink,” Biomed. Opt. Express 5(7), 2037–2053 (2014). 10.1364/BOE.5.002037 25071947 PMC4102347

[85] Jacques S. L., “Optical properties of biological tissues: a review,” Phys. Med. Biol. 58(11), R37–R61 (2013). 10.1088/0031-9155/58/11/R37 23666068

[86] Tom L. Philip A. W. Paul H. C., “Optical properties of human skin,” J. Biomed. Opt. 17(9), 0909011 (2012). 10.1117/1.JBO.17.9.090901 23085902

[87] Farina A. Torricelli A. Bargigia I. Spinelli L. Cubeddu R. Foschum F. Jäger M. Simon E. Fugger O. Kienle A. Martelli F. Di Ninni P. Zaccanti G. Milej D. Sawosz P. Kacprzak M. Liebert A. Pifferi A., “In-vivo multilaboratory investigation of the optical properties of the human head,” Biomed. Opt. Express 6(7), 2609–2623 (2015). 10.1364/BOE.6.002609 26203385 PMC4505713

[88] Sudakou A. Yang L. Wabnitz H. Wojtkiewicz S. Liebert A., “Performance of measurands in time-domain optical brain imaging: depth selectivity versus contrast-to-noise ratio,” Biomed. Opt. Express 11(8), 4348–4365 (2020). 10.1364/BOE.397483 32923048 PMC7449735

[89] Selb J. Ogden T. M. Dubb J. Fang Q. Boas D. A., “Comparison of a layered slab and an atlas head model for Monte Carlo fitting of time-domain near-infrared spectroscopy data of the adult head,” J. Biomed. Opt. 19(1), 016010 (2014). 10.1117/1.JBO.19.1.016010 24407503 PMC3886581

[90] Sato C. Shimada M. Tanikawa Y. Hoshi Y., “Estimating the absorption coefficient of the bottom layer in four-layered turbid mediums based on the time-domain depth sensitivity of near-infrared light reflectance,” J. Biomed. Opt. 18(9), 097005 (2013). 10.1117/1.JBO.18.9.097005 24057194

[91] Feng J. Zhang W. Li Z. Jia K. Jiang S. Dehghani H. Pogue B. W. Paulsen K. D., “Deep-learning based image reconstruction for MRI-guided near-infrared spectral tomography,” Optica 9(3), 264–267 (2022). 10.1364/OPTICA.446576 35340570 PMC8952193

[92] Sassaroli A. Martelli F. Fantini S., “Perturbation theory for the diffusion equation by use of the moments of the generalized temporal point-spread function. III. Frequency-domain and time-domain results,” J. Opt. Soc. Am. A 27(7), 1723–1742 (2010). 10.1364/JOSAA.27.001723 PMC3429950 20596162

[93] Press W. H. Teukolsky S. A. Vetterling W. T. Flannery B. P., *Numerical Recipes 3rd Edition: The Art of Scientific Computing* (Cambridge University Press, 2007).

[94] Ostojic D. Kleiser S. Nasseri N. Isler H. Andresen B. Wabnitz H. Karen T. Scholkmann F. Wolf M., “In vitro comparisons of near-infrared spectroscopy oximeters: impact of slow changes in scattering of liquid phantoms,” Adv. Exp. Med. Biol. 1072, 375–379 (2018). 10.1007/978-3-319-91287-5_60 30178374

